# Hoxa9 and Meis1 Cooperatively Induce Addiction to Syk Signaling by Suppressing miR-146a in Acute Myeloid Leukemia

**DOI:** 10.1016/j.ccell.2017.03.001

**Published:** 2017-04-10

**Authors:** Sebastian Mohr, Carmen Doebele, Federico Comoglio, Tobias Berg, Julia Beck, Hanibal Bohnenberger, Gabriela Alexe, Jasmin Corso, Philipp Ströbel, Astrid Wachter, Tim Beissbarth, Frank Schnütgen, Anjali Cremer, Nadine Haetscher, Stefanie Göllner, Arefeh Rouhi, Lars Palmqvist, Michael A. Rieger, Timm Schroeder, Halvard Bönig, Carsten Müller-Tidow, Florian Kuchenbauer, Ekkehard Schütz, Anthony R. Green, Henning Urlaub, Kimberly Stegmaier, R. Keith Humphries, Hubert Serve, Thomas Oellerich

**Affiliations:** 1 Department of Medicine II, Hematology/Oncology, Goethe University, Theodor-Stern-Kai 7, 60590 Frankfurt, Germany; 2 Department of Haematology, University of Cambridge, Hills Road, Cambridge CB2 0XY, UK; 3 Cambridge Institute for Medical Research, Wellcome Trust/MRC Stem Cell Institute, Cambridge CB2 0XY, UK; 4 German Cancer Research Center and German Cancer Consortium, 69120 Heidelberg, Germany; 5 Chronix Biomedical, Goetheallee 8, 37073 Göttingen, Germany; 6 Institute of Pathology, University Medical Center Göttingen, Robert-Koch-Straße 40, 37073 Göttingen, Germany; 7 Department of Pediatric Oncology, Dana-Farber Cancer Institute and Boston Children's Hospital, Boston, MA 02115, USA; 8 Broad Institute, Cambridge, MA 02142, USA; 9 Bioanalytical Mass Spectrometry Group, Max Planck Institute for Biophysical Chemistry, Am Fassberg 11, 37077 Göttingen, Germany; 10 Institute of Medical Statistics, University Medical Center Göttingen, Humboldtallee 32, 37073 Göttingen, Germany; 11 Department of Hematology and Oncology, University of Halle, Ernst-Grube-Street 40, 06120 Halle, Germany; 12 Department of Internal Medicine III, University Hospital of Ulm, Albert-Einstein-Allee 23, 89081 Ulm, Germany; 13 Department of Clinical Chemistry and Transfusion Medicine, Institute of Biomedicine, Sahlgrenska Academy at University of Gothenburg, Su sahlgrenska, 41345 Gothenburg, Sweden; 14 Department of Biosystems Science and Engineering, Eidgenössische Technische Hochschule (ETH) Zurich, 4058 Basel, Switzerland; 15 Institute for Transfusion Medicine and Immunohematology, Goethe University, Sandhofstraße 1, 60590 Frankfurt, Germany; 16 Bioanalytics, Georg August University, Robert-Koch-Straße 40, 37073 Göttingen, Germany; 17 Terry Fox Laboratory, British Columbia Cancer Agency, 675 West 10th Avenue, Vancouver, BC V5Z 1L3, Canada; 18 Department of Medicine, University of British Columbia, Vancouver, BC V5Z 1M9, Canada

**Keywords:** leukemia, signal transduction, microRNA, Syk, Hox genes, PU.1

## Abstract

The transcription factor Meis1 drives myeloid leukemogenesis in the context of *Hox* gene overexpression but is currently considered undruggable. We therefore investigated whether myeloid progenitor cells transformed by Hoxa9 and Meis1 become addicted to targetable signaling pathways. A comprehensive (phospho)proteomic analysis revealed that Meis1 increased Syk protein expression and activity. Syk upregulation occurs through a Meis1-dependent feedback loop. By dissecting this loop, we show that Syk is a direct target of miR-146a, whose expression is indirectly regulated by Meis1 through the transcription factor PU.1. In the context of Hoxa9 overexpression, Syk signaling induces Meis1, recapitulating several leukemogenic features of Hoxa9/Meis1-driven leukemia. Finally, Syk inhibition disrupts the identified regulatory loop, prolonging survival of mice with Hoxa9/Meis1-driven leukemia.

## Highlights

• Meis1 regulates expression and activation of Syk in Hoxa9-driven leukemia
• Syk upregulation in Hoxa9/Meis1-driven leukemia is mediated by miR-146a
• Increased Syk activity induces a Meis1 transcriptional program
• In vivo, Meis1 sensitizes Hoxa9-driven leukemia to Syk inhibition

## Graphical Abstract

**Figure:**
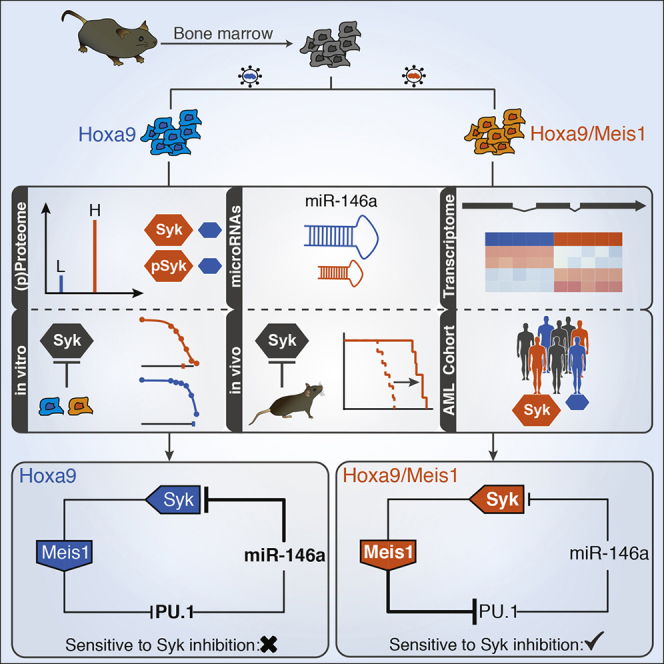

## Teaser

Mohr et al. identify a Meis1-dependent regulatory loop involving PU.1, miR-146a, and Syk, resulting in the addiction to Syk activity in Hoxa9/Meis1-transformed myeloid progenitor cells. Syk inhibition disrupts the regulatory loop and prolongs survival of mice with Hoxa9/Meis1-driven acute myeloid leukemia.

## Significance

**Overexpression of the *Hox* and *Meis1* genes triggers leukemogenesis, is associated with high-risk acute myeloid leukemia (AML) and currently cannot be targeted by drugs. Through the integration of a multi-omics approach with functional analyses we elucidated the molecular mechanism of Meis1 function and identified a Meis1-dependent regulatory feedback loop involving PU.1, miR-146a, and Syk. Transformation of myeloid progenitors with Hoxa9 and Meis1 induced addiction to Syk activity, and Syk itself induced Meis1 expression and a Meis1 transcriptional program. Hence, our study identifies Syk as a key regulator of Hoxa9/Meis1-driven AML and places it as a prime candidate for the clinical testing of Syk inhibitors in AML treatment.**

## Introduction

Acute myeloid leukemia (AML) is an aggressive neoplastic disease characterized by enhanced proliferation, blocked differentiation, and dysregulated apoptosis. AML appears to be driven by cell populations exhibiting extensive self-renewal properties, known as leukemia stem cells (LSCs). Despite an increased understanding of the genetic mutations driving the development of AML, the molecular processes that govern these self-renewal properties remain elusive (The Cancer Genome Atlas Research Network, 2013).

A large body of data implicates Hox genes in this process (Argiropoulos and Humphries, 2007). A central role for Hox genes in AML is supported by the frequently elevated Hox gene expression in AML cells (Afonja et al., 2000, Kawagoe et al., 1999, Lawrence et al., 1999). Hox gene overexpression is associated with genetically defined AML subgroups. Subsets of AML with favorable genetic features, such as core-binding factor leukemias and PML-RARα-positive leukemias, express low levels of Hox genes (Drabkin et al., 2002, Lawrence et al., 1999, Valk et al., 2004). In contrast, unfavorable genetic alterations, such as mixed-lineage leukemia (MLL) fusions (for instance MLL-AF9 and MLL-ENL) exhibit their transforming capacity largely through upregulation of Hox genes (Krivtsov and Armstrong, 2007, Muntean and Hess, 2012).

Among *Hox* genes, the Abd-B-type *Hox* genes (especially *Hoxa9*) are central regulators of the primitive hematopoietic compartment. *Hoxa9* is preferentially expressed in primitive hematopoietic cells and is downregulated during differentiation (Pineault et al., 2002, Sauvageau et al., 1994). A number of overexpression studies have also shown that certain *Hox* genes and *Hox* gene fusions have the ability to promote expansion of primitive hematopoietic cells (Ohta et al., 2007, Sauvageau et al., 1995). Similarly, *Hoxa9* enhances hematopoietic stem cell regeneration in vivo, ultimately leading to the development of leukemia, albeit with a long latency (Thorsteinsdottir et al., 2002).

Meis1 is another critical regulator of LSCs that is often overexpressed in Hox-gene-driven leukemia (Kawagoe et al., 1999, Lawrence et al., 1999). Although Meis1 alone is unable to promote self-renewal, it plays a role in establishing LSC potential in MLL-rearranged leukemias (Wong et al., 2007). Moreover, when combined with overexpression of a *Hox* gene or the *NUP98-Hox* fusion gene, overexpression of *Meis1* leads to a massive acceleration of leukemia development (Kroon et al., 1998, Pineault et al., 2004). Gene expression studies have identified a number of Meis1 target genes, some of which are critical for leukemogenesis (Argiropoulos et al., 2008, Kuchenbauer et al., 2008, Kuchenbauer et al., 2011, Wang et al., 2006). One such target is the tyrosine kinase *Flt3*, which in combination with a *NUP98-Hox* fusion gene accelerates leukemogenesis (Palmqvist et al., 2006, Wang et al., 2005). However, Flt3 appears to be dispensable for Meis1-induced leukemic transformation (Argiropoulos et al., 2008, Morgado et al., 2007).

While several studies have focused on Meis1 target genes, only a few have examined the intracellular signaling pathways affected by Meis1 overexpression. These studies showed that Meis1 enhances signaling through Akt and Erk (Argiropoulos et al., 2008) and activates the MAP kinase and PI3K/Akt pathways (Gibbs et al., 2012), and that activation of Wnt signaling is required for transformation of committed myeloid progenitors by Hoxa9 and Meis1 (Wang et al., 2010).

However, our understanding of the interplay between Hoxa9- and Meis1-regulated genes, its impact on signaling pathways, and its functional consequences remains limited. Because Hoxa9 and Meis1 overexpression is frequent in high-risk AML (Drabkin et al., 2002, Heuser et al., 2009, Zangenberg et al., 2009), and because both factors are currently considered undruggable, we set out to elucidate the effects of combined Hoxa9/Meis1 overexpression on intracellular signaling and to investigate whether cells transformed by Hoxa9 and Meis1 become addicted to targetable signaling processes.

To this end, we integrated a multi-omics approach including mass-spectrometry-based proteomics, phosphoproteomics, and transcriptome profiling, with targeted functional cell-based and in vivo analyses.

## Results

### Meis1 Induces Syk Signaling in Hoxa9-Overexpressing Myeloid Progenitors

**Figure 1:**
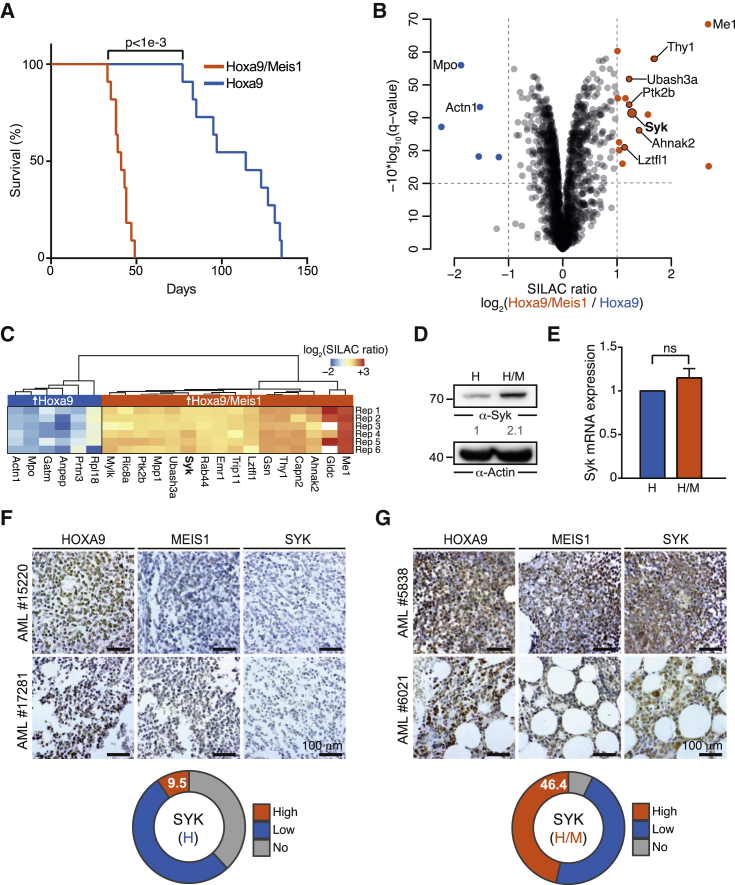
Meis1 Increases Syk Protein Levels in Hoxa9-Driven Leukemia (A) Kaplan-Meier survival curves of mice transplanted with either H- or H/M-transformed myeloid progenitor cells (n = 11). The p value is from a Mantel-Cox test. (B) Volcano plot relating q values for differential protein expression to average normalized SILAC ratios from six biological replicates. Blue (higher expression in H cells) and orange (higher expression in H/M cells) dots indicate significantly regulated proteins (q < 0.01). (C) Heatmap of SILAC ratios for significantly differentially expressed proteins in H and H/M cells across the six biological replicates. (D) Syk protein expression in H and H/M cells by immunoblotting. Actin was used as loading control for relative protein quantification. (E) Relative *Syk* mRNA expression as measured by qPCR, normalized to *GAPDH* expression (mean ± SD, n = 3); ns, not significant (two-sided unpaired t test). (F and G) Immunohistochemical staining of HOXA9, MEIS1, and SYK in bone marrow biopsies from patients with AML. SYK expression levels were analyzed in 21 AML cases with high HOXA9 expression (F) and 28 cases with high HOXA9/MEIS1 expression (G). Proportions of SYK expression levels as determined by two independent pathologists using a three-stage staining score are shown. See also Figure S1, Tables S1, and S2.

To elucidate the molecular mechanisms underlying the contribution of Meis1 to leukemogenesis, we employed a retroviral transplantation model in which lineage-depleted mouse bone marrow cells were transduced with an MSCV-Hoxa9-PGK-neo construct, alone or in combination with an MSCV-Meis1-IRES-YFP construct that induced a 22-fold overexpression of *Meis1* (Figure S1A). As reported by others, the transformed cells could be cultured in vitro in the presence of interleukin-3 (IL-3)/IL-6/stem cell factor (SCF) and expressed the expected immunophenotype characterized by the myeloid markers Mac-1 and Gr-1 as well as c-Kit (Figure S1B) (Pineault et al., 2005, Wang et al., 2005). When transplanted into irradiated recipient mice, cells transduced with Hoxa9 (H) or Hoxa9/Meis1 (H/M) gave rise to leukemia resulting in a median overall survival of 114 and 41 days, respectively (p < 0.001; Figure 1A). This difference in survival is in accordance with previously published results and reflects the aggressiveness of Hoxa9/Meis1-driven AML observed in patients (Kroon et al., 1998).

Because mRNA expression levels only moderately correlate with actual protein levels (Schwanhausser et al., 2011, Vogel and Marcotte, 2012), we set out to analyze the consequences of Meis1 expression on the cellular proteome by combining stable isotope labeling by amino acids in cell culture (SILAC) and mass spectrometry (Figure S1C). This quantitative protein expression analysis of H and H/M cells allowed the reproducible identification and quantification of 1,810 proteins in at least four out of six biological replicates (Table S1).

Interestingly, two tyrosine kinases, focal adhesion kinase 2 (Ptk2b) and spleen tyrosine kinase (Syk), were among the most upregulated proteins in H/M cells (Figures 1B and 1C). Overexpression of Syk in H/M cells was confirmed by immunoblotting (Figure 1D). Notably, real-time qPCR and RNA sequencing (RNA-seq) indicated that *Syk* was not upregulated at the mRNA level in H/M cells (qPCR fold change 1.15; RNA-seq fold change 1.13; Figure 1E), thus explaining why several independent RNA expression analyses did not link *Syk* to Meis1 (Argiropoulos et al., 2008, Argiropoulos et al., 2010, Huang et al., 2012, Wang et al., 2005, Wang et al., 2006, Wilhelm et al., 2011). To test whether the combined H/M overexpression is also associated with enhanced Syk protein expression in primary human AML samples, we performed immunohistochemical (IHC) analyses for HOXA9, MEIS1, and SYK on a cohort of 115 AML cases (Table S2). We found overexpression of HOXA9 alone in a total of 21 cases and overexpression of both HOXA9 and MEIS1 in 28 cases, with only one Flt3-ITD-positive patient. Increased SYK expression was significantly more frequent (>4 times, p = 0.01, Fisher's exact test) in samples with a high expression of HOXA9 and MEIS1 (46.4%) than in HOXA9-overexpressing samples (9.5%) (Figures 1F and 1G). This frequency is also >2 times higher than in HOXA9 and MEIS1 double-negative samples (22.7%) (Figure S1D).

Hence, combined overexpression of Hoxa9 and Meis1 leads to upregulation of Syk at the post-transcriptional level, and elevated SYK expression is associated with HOXA9/MEIS1 overexpression in human AML samples.

**Figure 2:**
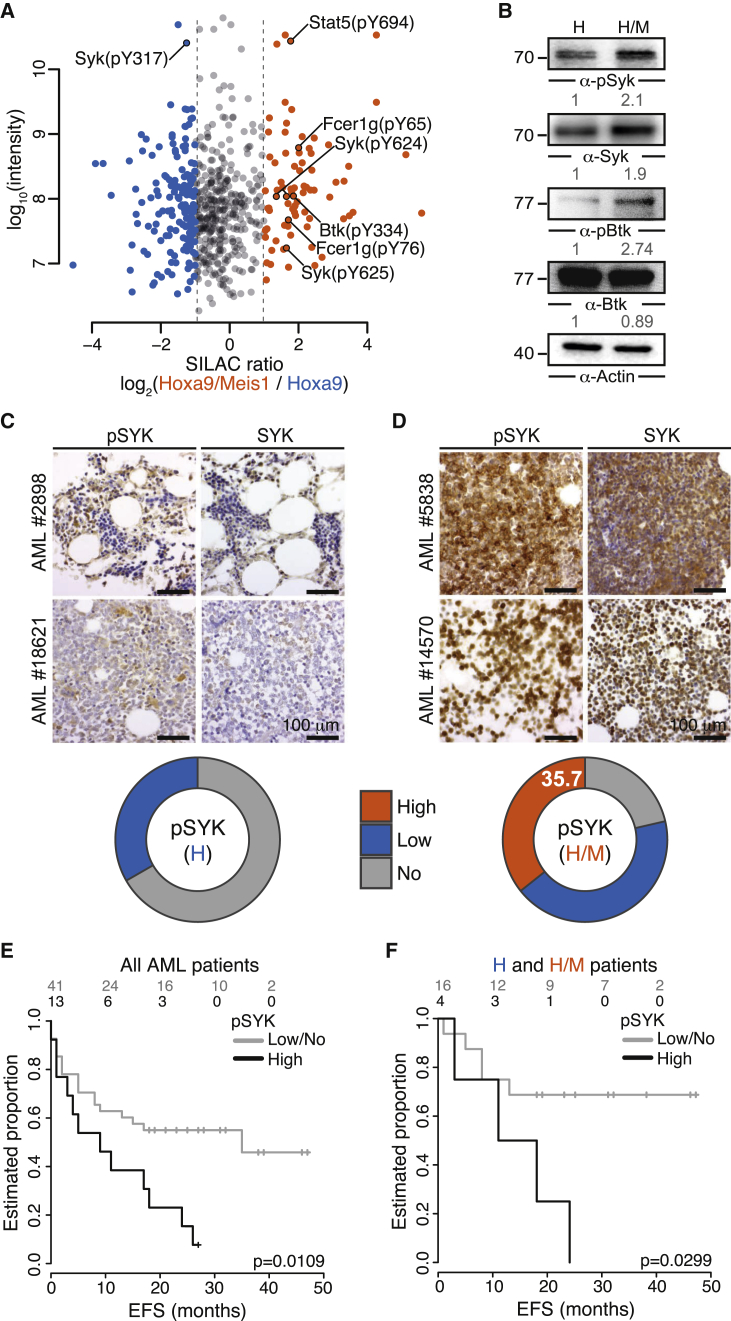
Enhanced Syk Signaling in H/M Cells (A) Intensities of peptide peaks versus average normalized SILAC ratios for p-sites identified by a mass-spectrometric pYome analysis in two biological replicates. Blue and orange dots indicate p-sites upregulated in H and H/M cells, respectively. Selected p-sites are labeled. (B) Validation of selected differential tyrosine phosphorylation events in H and H/M cells by immunoblotting. Actin was used as loading control for relative protein quantification. (C and D) Immunohistochemical staining of phospho-SYK (pY348) and SYK in bone marrow biopsies from AML patients. pSYK levels were analyzed in 21 human AML cases with high HOXA9 expression (C) and 28 cases with high HOXA9/MEIS1 expression (D). Proportions of pSYK levels as determined by two independent pathologists using a three-stage staining score are shown. (E and F) Kaplan-Meier survival analysis for event-free survival (EFS) in which all AML patients with complete clinical profiles (E) or H and H/M patients only (F) were grouped by pSYK expression. The number of patients at risk belonging to each category is shown. The p value is from a Mantel-Cox test. See also Figure S2, Tables S1, and S2.

The deregulated expression of kinases prompted us to examine the global impact of Meis1 overexpression on intracellular signaling by a mass spectrometry-based phosphoproteomic analysis of H and H/M cells. The analysis was performed after enrichment for phosphorylated tyrosine residues (pYome) and separately after enrichment for phosphoserine, threonine, and tyrosine residues (global phosphoproteome, GPome) (Figure S2A). We identified and quantified a total of 584 class I phosphorylation events (p-events with a localization probability >75%) in the pYome and 3,305 class I p-events in the GPome, of which 236 and 297 were differentially regulated between H and H/M cells, respectively (Figures 2A and S2B; Table S1). Notably, this analysis revealed enhanced phosphorylation of the Syk-activating tyrosines Y624/625 and dephosphorylation of the inhibitory tyrosine Y317 in H/M cells, suggesting enhanced Syk signaling in H/M cells. This result is furthermore supported by an enhanced tyrosine phosphorylation of Stat5 and Btk, two effectors known to be activated by Syk in AML and B cells, respectively (Carnevale et al., 2013, Oellerich et al., 2013) (Figure 2A). Differential phosphorylation of Syk and Btk was confirmed by immunoblotting (Figure 2B).

As Meis1 not only enhanced Syk protein expression, but also increased its activation-inducing tyrosine phosphorylation in our model system, we set out to validate this finding in our cohort of primary AML samples. Therefore, we performed IHC analyses for phosphorylated SYK (pY348, a Syk-activating p-site) in the 21 AML cases overexpressing HOXA9 alone and in the 28 cases overexpressing both HOXA9 and MEIS1 (Figures 2C and 2D). This analysis revealed a significant association between strong SYK phosphorylation and HOXA9/MEIS1 overexpression (35.7% of H/M samples) compared with samples in which only HOXA9 was overexpressed (0% of H samples; p < 0.003, Fisher's exact test) or double-negative samples (13.6%; p = 0.024) (Figures 2C, 2D, and S2C). Moreover, high SYK phosphorylation correlates with poor event-free and relapse-free survival in the subset of AML patients with complete clinical profiles within our cohort, both with and without stratification for H and H/M expression (Figures 2E, 2F, S2D, and S2E). Together, these results indicate a strong association of MEIS1 overexpression with upregulation and activation of SYK in AML.

### Enhanced Syk Activation Is Partly Dependent on Integrin Beta 3

**Figure 3:**
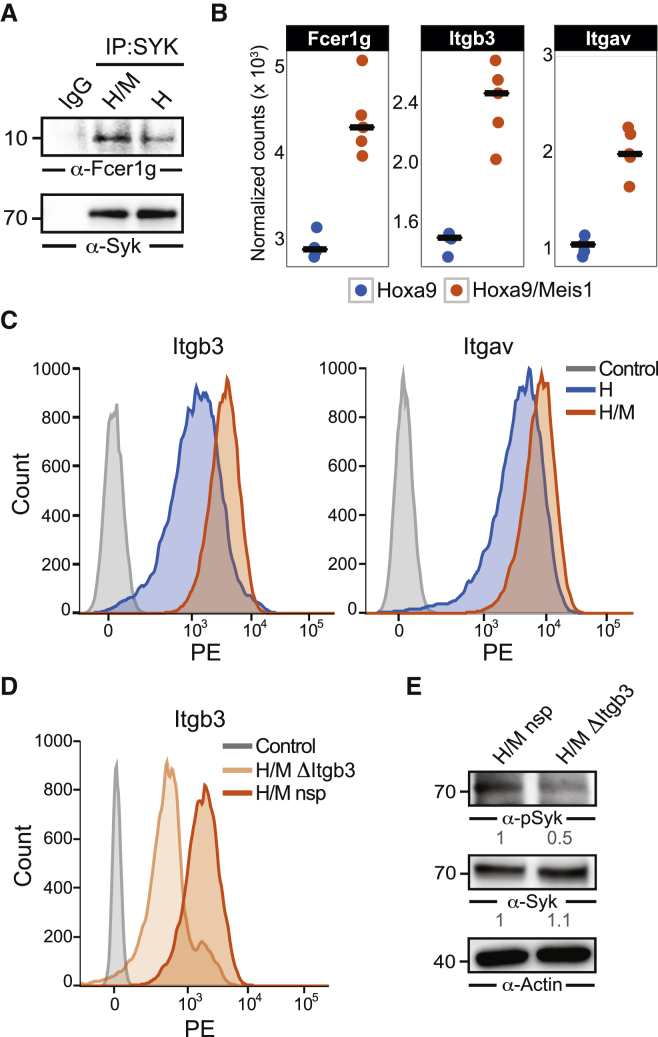
Syk Phosphorylation Is Partly Dependent on Integrin Beta 3 (A) Co-immunoprecipitation of Fcer1g and Syk from H and H/M cells. (B) *Fcer1g*, *Itgb3*, and *Itgav* expression estimated by normalized RNA-seq counts. (C) Itgb3 and Itgav cell surface expression in H and H/M cells measured by flow cytometry. Unstained cells were used as controls. (D) Itgb3 cell surface expression in H/M cells transduced with either a lentiviral non-specific (nsp) control CRISPR or a CRISPR targeting *Itgb3* (ΔItgb3). (E) Corresponding (p)Syk expression determined by immunoblotting. Actin was used as loading control for relative protein quantification.

We next investigated potential mechanisms of Syk activation in H/M cells. Syk activation requires docking to phosphorylated immunoreceptor tyrosine-based activation motifs (ITAMs) (Kulathu et al., 2009). Interestingly, our pYome analysis revealed increased ITAM phosphorylation of the common Fcγ-chain Fcer1g in H/M cells (Figure 2A). Fcer1g is an intracellular signaling module that associates with Fc receptors and integrins (Humphrey et al., 2005). While depletion of Fc receptors does not affect viability and proliferation of AML cells, integrin beta 3 (Itgb3) is required for leukemogenesis (Miller et al., 2013, Oellerich et al., 2013). Notably, Fcer1g interacts with Syk in H cells and, in line with enhanced ITAM phosphorylation, this interaction is stronger in H/M cells (Figure 3A). In addition, Meis1 overexpression in H cells increased transcript levels of Fcer1g, Itgb3, and its heterodimeric partner integrin alpha v (Itgav), and upregulated Itgb3/Itgav expression on the cell surface (Figures 3B and 3C). To test whether increased Itgb3 cell surface expression translates into increased Syk activity, we knocked out Itgb3 using CRISPR/Cas9 by transducing H/M cells with a lentiviral Itgb3 CRISPR construct (ΔItgb3) (Figure 3D). Itgb3 knockout led to a 50% reduction in activatory Syk phosphorylation (pY525/526) in a polyclonal cell population (Figure 3E), indicating that enhanced Syk activation in H/M cells depends, at least in part, on Itgb3.

### Syk Expression Is Regulated by miR-146a

**Figure 4:**
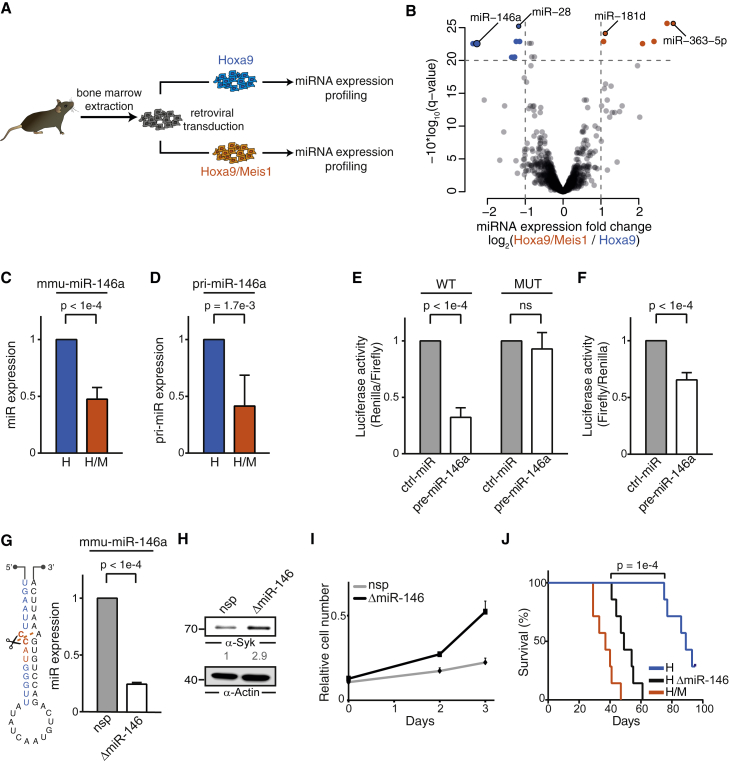
Syk Is a Direct Target of miR-146a (A) Schematic workflow of the miRNA expression analysis in H- and H/M-transformed myeloid progenitors. (B) Volcano plot relating q values for differential miRNA expression between H and H/M cells to average miRNA expression fold-changes from three biological replicates. Blue (higher expression in H cells) and orange (higher expression in H/M cells) dots indicate significantly regulated miRNAs (q < 0.01). (C and D) Relative mmu-miR-146a expression (C) and pri-miR-146a expression (D) in H/M versus H cells, measured by qPCR and normalized to *sno202* and *GAPDH* expression, respectively (mean ± SD, n = 3). The p values are from a two-sided unpaired t test. (E) Luciferase assay validating binding of miR-146a to the predicted target sites within the 3′ UTR of Syk (mean ± SD, n = 4); WT, predicted miR-146a target sequence; MUT, mutated version thereof. The p values are from a two-sided unpaired t test. ns, not significant. (F) Luciferase assay validating binding of miR-146a to the full-length Syk 3′ UTR (mean ± SD, n = 4). The p value is from a two-sided unpaired t test. (G) Left, secondary structure of mmu-miR-146 as predicted by RNAfold (Lorenz et al., 2011). The CRISPR/Cas9 cleavage site is indicated. Right, relative expression of miR-146a, measured by qPCR and normalized to *sno202* expression, in H cells transduced with either a lentiviral non-specific (nsp) control CRISPR or a CRISPR targeting *miR-146* (ΔmiR-146) (mean ± SD, n = 3). The p value is from a two-sided unpaired t test. (H) Corresponding Syk protein expression by immunoblotting. Actin was used as loading control for relative protein quantification. (I) Cell-proliferation curves for H cells transduced with either a lentiviral non-specific (nsp) control CRISPR or a CRISPR targeting *miR-146* (ΔmiR-146) (mean ± SD, n = 3). (J) Kaplan-Meier survival curves of mice transplanted with H or H/M cells transduced with a lentiviral non-specific (nsp) control CRISPR, or with H cells transduced with a CRISPR targeting *miR-146* (ΔmiR-146) (n = 7). The p value is from a Mantel-Cox test. See also Figure S3.

Because the upregulation of Syk in H/M cells was only detectable at the protein but not at the mRNA level (Figures 1B–1E), and because no differences were detected in the proteasomal degradation of Syk (data not shown), we reasoned that microRNAs (miRNAs) might be involved in the regulation of Syk. To test this hypothesis, we globally profiled miRNA expression in H and H/M cells (Figure 4A). This analysis identified eight significantly downregulated miRNAs in H/M cells potentially responsible for the observed upregulation of Syk (Figures 4B and S3A). To refine our candidate list, we retained only those miRNAs that were predicted to target Syk by *Targetscan* (Agarwal et al., 2015). The algorithm identified two predicted binding sites for miR-146a in the 3′ UTR of Syk. A significant downregulation of mmu-miR-146a and pri-miR-146a in H/M relative to H cells was further confirmed by qPCR (Figures 4C and 4D).

To experimentally validate targeting of Syk by miR-146a, we performed luciferase assays using two reporter constructs, one containing two copies of both predicted miR-146a binding sites (or mutated versions as controls; Figure 4E) and one containing the full-length Syk 3′ UTR (Figure 4F). Overexpression of miR-146a precursor (pre-miR-146a) decreased luciferase activity in lysates of HEK293T cells transfected with the construct containing the miR-146a target sites or the Syk 3′ UTR, but had no effect on the construct with mutated binding sites (Figures 4E and 4F). This result indicates that Syk is a direct miR-146a target.

To further test whether miR-146a affects Syk expression, we knocked out miR-146 using CRISPR/Cas9 by transducing H cells with a lentiviral miR-146-specific CRISPR construct (ΔmiR-146) that reduced miR-146a expression by 75% in a polyclonal cell population (Figure 4G) or isolated myeloid progenitor cells from B6/miR-146a^−/−^ mice and transduced them with Hoxa9. The CRISPR-mediated knockout of miR-146 led to a 2.9-fold increase in the protein expression of Syk (Figure 4H), increased cell proliferation (Figures 4I and S3B), reduced apoptosis (Figure S3C), and enhanced c-Kit expression (Figure S3D), mirroring the phenotype of H/M cells. Finally, mice transplanted with miR-146 knockout H cells exhibited accelerated leukemia development compared with mice transplanted with H cells (Figure 4J).

In summary, our data strongly indicate that upregulation of Syk in H/M cells is mediated by downregulation of miR-146a.

### Meis1 Influences miR-146a Expression through Downregulation of PU.1

**Figure 5:**
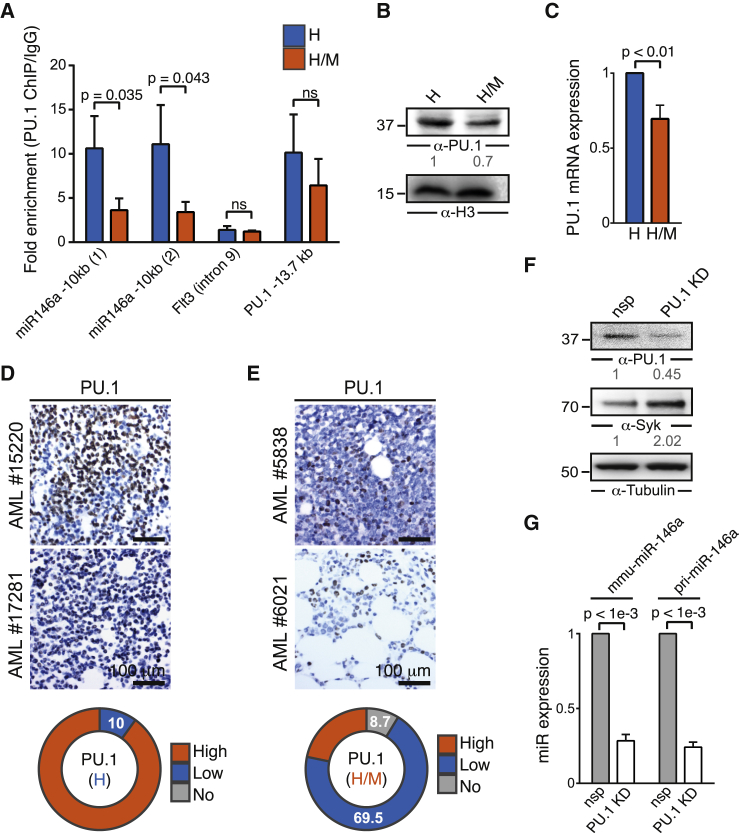
Meis1 Downregulates miR-146a through PU.1 (A) Fold enrichment of PU.1 binding over IgG control as measured by ChIP-qPCR in H and H/M cells (mean ± SD, n = 3). The miR-146a −10 kb region spans the transcription start site of the miR-146a host gene; ns, not significant. (B) PU.1 protein expression in H and H/M cells by immunoblotting. Histone H3 was used as loading control for relative protein quantification. (C) Relative *PU.1* mRNA expression in H versus H/M cells measured by qPCR and normalized to *GAPDH* expression (mean ± SD, n = 3). (D and E) Immunohistochemical staining of PU.1 in bone marrow biopsies from patients with AML. PU.1 expression levels were analyzed in 21 AML cases with high HOXA9 expression (D) and 28 cases with high HOXA9/MEIS1 expression (E). Proportions of PU.1 expression levels as determined by two independent pathologists using a three-stage staining score are shown. (F) PU.1 and SYK protein expression by immunoblotting in H cells transfected with either a control shRNA (nsp) or an shRNA targeting PU.1 (KD). Tubulin was used as loading control for relative protein quantification. (G) mmu-miR-146a and pri-miR-146a expression as measured by qPCR after PU.1 knockdown (KD) relative to control shRNA (nsp) (mean ± SD, n = 4). The p values are from a two-sided unpaired t test. See also Figure S4 and Table S2.

**Figure 6:**
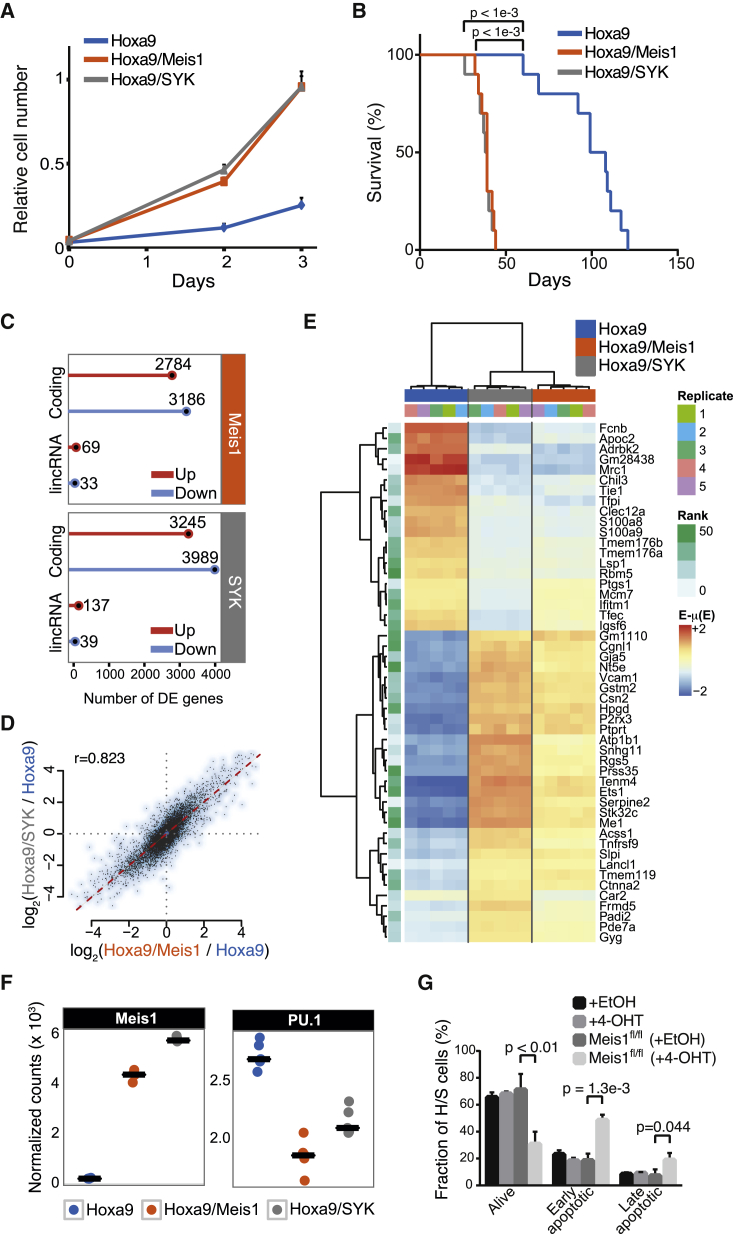
Syk Overexpression Mimics the Leukemogenic Meis1 Transcriptional Program in Hoxa9-Driven Leukemia (A) Proliferation curves for H, H/M and H/S cells (mean ± SD, n = 4). (B) Kaplan-Meier survival curves of mice transplanted with either H (n = 9), H/M (n = 10) or H/S (n = 11) cells. The p values are from a Mantel-Cox test. (C) Summary of differentially expressed (DE) protein-coding genes and lincRNAs (Benjamini-Hochberg adjusted p value ≤ 0.001, Wald test) in H-transformed myeloid progenitors upon overexpression of Meis1 (upper panel) and SYK (lower panel). (D) Gene expression correlation between H/M and H/S cells. Only genes that were significantly differentially expressed in at least one condition (Benjamini-Hochberg adjusted p value ≤ 10^−5^, likelihood ratio test) were considered. Correlation value (r) is Spearman's rank correlation coefficient. (E) Hierarchical clustering of the top 50 differentially expressed genes. Regularized log2 expression values are row-mean subtracted. (F) *Meis1* and *PU.1* expression estimated by normalized RNA-seq counts. Black lines denote the median. (G) Apoptosis analysis of H/S cells derived from either C57BL/6J mice or inducible Meis1 knockout mice, based on Annexin V/7-AAD staining (mean ± SD, n = 3). Cells were treated with either ethanol (EtOH, control) or 4-hydroxytamoxifen (4-OHT). The p values are from a two-sided unpaired t test. See also Figure S5.

Next, we investigated the molecular mechanism by which Meis1 downregulates miR-146a. No Meis1 binding site was found in the vicinity of the *miR-146a* locus in published Meis1 chromatin immunoprecipitation sequencing (ChIP-seq) profiles in myeloid cells (Heuser et al., 2011, Huang et al., 2012) (Figure S4A). However, miR-146a is known to be regulated by PU.1 (Spi1) in macrophages (Ghani et al., 2011). Therefore, we examined the binding of PU.1 to a previously identified PU.1 binding site located 10 kb upstream (−10 kb) of *miR-146a* by ChIP-qPCR. This region exhibits epigenomic features of an active promoter, including an enrichment for H3K4me3 and binding of RNA polymerase II in ENCODE data (Encode Project Consortium, 2012) (Figure S4B). We found that PU.1 binding to the −10 kb site was significantly reduced in H/M compared with H cells (Figure 5A), suggesting that decreased PU.1 binding might be responsible for the downregulation of *miR-146a*. Consistent with this finding, we also detected lower PU.1 protein and mRNA levels in H/M compared with H cells (Figures 5B, 5C, and 6F). In addition, an Integrated Motif Activity Response Analysis based on transcriptome profiles of H and H/M cells indicated decreased PU.1 activity in H/M relative to H cells (Figure S4C).

Moreover, we found a significant association between low or no PU.1 protein expression and high expression of HOXA9 and MEIS1 (78.2% compared with 10% for HOXA9-overexpressing samples, p < 9 × 10^−6^, Fisher's exact test) in our AML patient cohort (Figures 5D and 5E). Finally, a 55% knockdown of PU.1 in H cells reduced miR-146a expression and increased Syk protein levels (Figures 5F and 5G). Taken together, these data indicate that, by acting through PU.1, Meis1 indirectly influences the expression of miR-146a.

### Syk Overexpression Triggers a Meis1-Dependent Transcriptional Program

We next sought to characterize the functional consequences of Syk overexpression in the context of Hoxa9-driven leukemias. For this purpose, we examined the consequences of a lentiviral overexpression of human *SYK* (hSYK) in H cells in vitro and in vivo. Of note, Syk expression levels were comparable between H/M cells and cells overexpressing Hoxa9 and hSYK (H/S) (Figures S5A and S5B). hSYK overexpression resulted in enhanced cell proliferation rates in the presence of IL-3, IL-6, and SCF, mimicking the overexpression of Meis1 (Figure 6A). In addition, it enhanced the colony-formation capacity and replating efficiency of H cells in colony assays; both features suggest increased self-renewal (Figure S5C).

While Hoxa9 alone is sufficient to enable replating, both Meis1 and hSYK enhanced replating efficiency. This ability was significantly reduced by the Syk inhibitor R406, which decreased replating efficiency of H/M and H/S cells while moderately affecting colony formation and replating efficiency of H cells (Figure S5C).

We next investigated whether hSYK overexpression affected the leukemogenicity of H cells upon transplantation into lethally irradiated recipient mice. We found that the combination of Hoxa9 and hSYK significantly increased the aggressiveness of the leukemias compared with Hoxa9 alone (median of 38.5 versus 103.5 days; p < 0.001), with a median survival remarkably similar to that of Hoxa9/Meis1 (39 days; Figures 6B and S5P). The observed leukemias were classified as AML with a dense infiltration of leukemic blasts in the bone marrow, spleen, and liver, and leukemic blasts in the peripheral blood (Figures S5D–S5M). Leukemias induced by the combination of Hoxa9 with hSYK or Hoxa9 with Meis1 were characterized by lower leukocyte counts and a more pronounced anemia compared with Hoxa9 alone (Figure S5N). Immunophenotyping showed that the leukemic cells expressed c-Kit and the myeloid antigens Gr-1 and Mac-1, in agreement with previously published immunophenotypes of Hoxa9/Meis1-driven AML (Kroon et al., 1998). In addition, the frequency of c-Kit-positive cells was higher for Hoxa9 and Meis1, and for Hoxa9 and hSYK, compared with Hoxa9 alone (Figure S5O), suggesting a more immature phenotype of the developing leukemias.

SYK activation depends on the phosphorylation of Y348 and Y352 (Kulathu et al., 2009). To test whether the accelerated leukemia development exhibited by H/S cells is dependent on SYK activation, we transplanted H cells expressing either hSYK or a hSYK Y348F/Y352F double mutant into lethally irradiated recipient mice and monitored overall survival. Notably, hSYK double mutant abrogated the enhanced leukemogenicity of H/S cells (Figure S5P), indicating that SYK activation is necessary for this feature.

The striking phenotypic similarity between H/M and H/S cells led us to compare the transcriptional consequences of Meis1 and hSYK overexpression in Hoxa9-transformed myeloid progenitors by RNA-seq. By analyzing protein-coding and non-coding transcriptome compartments, we found that both Meis1 and hSYK profoundly alter the transcriptome of H cells, leading to the differential expression of thousands of protein-coding genes and >100 long intergenic non-coding RNAs (lincRNAs; Figures 6C, S6A, and S6B). Intriguingly, these transcriptional changes were highly correlated (*r* = 0.823) between H/M and H/S cells (Figure 6D), which share a common transcriptional signature (Figures 6E and S6B). Moreover, hSYK induced expression of *Meis1* to levels comparable with those in H/M cells (Figure 6F) and differentially expressed genes in H/M and H/S cells were similarly enriched for direct Meis1 binding to their promoter regions (Huang et al., 2012) (Figure S6C). Importantly, Meis1 expression is necessary for survival of H/S cells, as an inducible Meis1 knockout significantly affected H/S cell viability (Figures 6G and S6D).

Together, these results indicate that Meis1 and Syk regulate highly overlapping transcriptional programs and implicate Meis1 as an effector of Syk signaling to chromatin.

### Hoxa9/Meis1-Overexpressing Myeloid Progenitors Are Syk Dependent

To test whether the enhanced Syk signaling observed in H/M cells could be exploited therapeutically, we analyzed the effects of Syk inhibitors and of a small hairpin RNA (shRNA)-mediated knockdown of Syk in H/M cells, in vitro and in vivo. As shown above, the Syk inhibitor R406 significantly reduced colony-formation potential and replating efficiency in H/M cells (Figure S5C).

**Figure 7:**
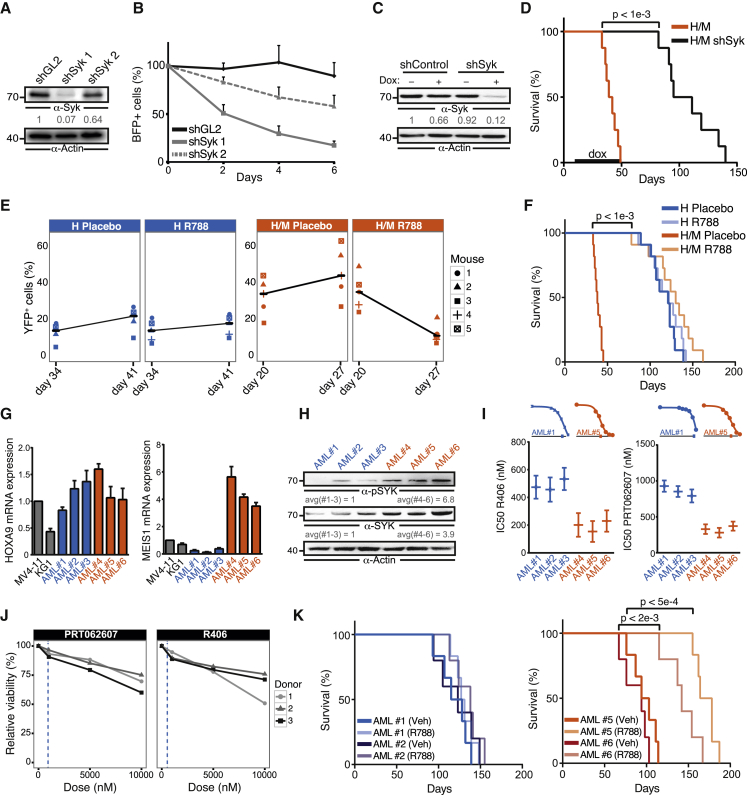
Meis1 Sensitizes Hoxa9-Driven Leukemia to Syk Inhibition (A) Syk protein expression in H/M cells transfected with either a control shRNA (GL2) or two shRNAs targeting Syk. Actin was used as loading control for relative protein quantification. (B) Percentage of BFP-positive shRNA-expressing cells relative to BFP-negative shRNA-negative cells at the times indicated (mean ± SD, normalized to day 0, n = 3). (C) Same as (A), before and after 5 days of doxycycline (dox) treatment in vivo. (D) Kaplan-Meier survival curves of mice transplanted with H/M cells and treated with doxycycline for 43 days to express non-specific control and Syk-specific shRNA (n = 8). The p value is from a Mantel-Cox test. (E) Percentage of YFP-positive cells from peripheral blood of mice transplanted with H (left) or H/M (right) cells after treating for 7 days with R788 or placebo. Measurements were taken at the indicated time points. The black line connects median values. (F) Kaplan-Meier survival curves of mice transplanted with either H or H/M cells and treated for 20 days with R788 or placebo (n = 11). The p value is from a Mantel-Cox test. (G) Relative *HOXA9* and *MEIS1* mRNA expression in MV4-11 and KG1 cell lines, and in patient-derived AML cells as measured by qPCR, normalized to *GAPDH* expression (mean ± SD, n = 3). (H) (p)SYK expression in the patient-derived AML cells in (G). Actin was used as loading control for relative protein quantification. avg, average. (I) Half maximal inhibitory concentration (IC_50_) for R406 (left) and PRT062607 (right) in patient-derived AML cells as determined by an Annexin V/7-AAD apoptosis assay. Cells were treated for 24 hr and DMSO was used as a control (n = 3). Representative dose-response curves for AML no. 1 (HOXA9 high, MEIS1 low) and AML no. 5 (HOXA9 high, MEIS1 high) are shown at the top. Ticks correspond to estimated IC_50_ values. (J) Relative viability of CD34^+^ bone marrow cells from healthy donors. Cells were treated with either R406 or PRT062607. Blue lines indicate the IC_50_ for both SYK inhibitors in H cells. (K) Kaplan-Meier survival curves of NSG mice transplanted with patient-derived AML cells indicated in (G) and treated for 14 days with R788 or vehicle (n = 6 for AML no. 1 and 5; n = 5 for AML no. 2 and 6). The p values are from a Mantel-Cox test. See also Figure S6.

We further examined the effect of Syk inhibition in H and H/M cells by monitoring the fate of individual cells and their progeny by time-lapse microscopy and single-cell tracking (Rieger et al., 2009). This allowed us to track hundreds of H and H/M cells over more than 50 hr in real time and to record their history across cell generations. Our analysis revealed a significant increase in cell death in R406-treated H/M cells compared with DMSO-treated cells, whereas H cells were not significantly affected (Figure S6E). These results are not mediated by off-target effects of R406, as knocking down Syk in H/M cells with a doxycycline-inducible lentiviral shRNA resulted in decreased cell viability in vitro (Figures 7A and 7B). In addition, we knocked down Syk in vivo by transplanting mice with cells that were either transduced with the doxycycline-inducible lentiviral Syk shRNAs or with control shRNAs and treating them with doxycycline for 43 days. Knockdown of Syk prolonged the survival of mice transplanted with H/M cells from a median time of 40.5 days in controls to a median of 103 days (p < 0.001) (Figures 7C and 7D). Furthermore, we treated mice transplanted with H/M or H cells with the oral Syk inhibitor R788, a prodrug of R406. Seven days of treatment with R788 reduced the percentage of leukemic cells in mice transplanted with H/M cells by more than 70% on average, while barely affecting the level of H cells (Figure 7E). Treatment with R788 for 20 days significantly prolonged the survival of mice transplanted with H/M cells from a median of 38 days to a median of 130 days (p < 0.001; Figure 7F). No significant effect was observed in mice transplanted with H cells.

Given the pronounced sensitivity of Hoxa9/Meis1-transformed mouse hematopoietic progenitors to Syk inhibition, we examined whether this effect can also be recapitulated in primary human AML samples. For this purpose, we considered three AML samples exhibiting strong *HOXA9* expression and weak *MEIS1* expression, and compared them with three samples expressing both genes at high levels (Figure 7G). Notably, none of these samples harbored activating mutations in *FLT3*. We found that AML samples expressing high levels of both *HOXA9* and *MEIS1* exhibited increased expression of SYK and pSYK, and were more sensitive to the SYK inhibitors PRT062607 and R406 compared with samples with weak *MEIS1* expression (Figures 7H–7I). Importantly, Syk inhibition did not affect the viability of CD34^+^ progenitor cells isolated from healthy donors (Figure 7J). Finally, Syk inhibition significantly prolonged survival of NSG mice transplanted with patient-derived AML cells overexpressing HOXA9 and MEIS1, with no significant difference for HOXA9 alone (Figure 7K).

In summary, our results demonstrate that enhanced Syk signaling in the presence of Meis1 represents a regulatory feedback mechanism of leukemogenesis in Hoxa9-driven AML that renders these cells sensitive to Syk inhibition.

## Discussion

Several studies characterized gene expression signatures and individual target genes regulated by Hoxa9 and Meis1. Among those, only a few at most partially recapitulate the oncogenic effects of Hoxa9 and Meis1.

In this work, we employed quantitative mass spectrometry to study proteomic and phosphoproteomic changes induced by Meis1 overexpression, identify Meis1-regulated proteins and signaling pathways, and investigate their therapeutic potential. By this approach we identified upregulation and activation of Syk by Meis1 as a key leukemogenic mechanism in a Hoxa9-driven mouse model system and in a subset of human AMLs.

Syk was originally described as a signaling mediator downstream of the B cell antigen receptor, but it has also been identified as a drug target for the treatment of AML (Hahn et al., 2009). In addition, Syk has been shown to be activated by integrin signaling, to phosphorylate STAT5 in AML (Miller et al., 2013, Oellerich et al., 2013) and to cooperate with FLT3-ITD during the induction and maintenance of myeloid leukemias (Puissant et al., 2014). Moreover, SYK Y323 phosphorylation in AML has recently been correlated with an unfavorable prognosis (Boros et al., 2015), and activatory SYK phosphorylation (pY348) correlates with poor event-free and relapse-free survival in our AML patient cohort.

Our results indicate that Syk protein levels, but not mRNA levels, are upregulated upon overexpression of Meis1 through a post-transcriptional mechanism. By analyzing Meis1-dependent miRNA expression changes, we found that Meis1 downregulates miR-146a, which in turn regulates Syk expression post-transcriptionally. We have thereby identified a regulatory link between miR-146a and Syk that is indirectly orchestrated by Meis1. Similar regulatory paradigms may have implications for cell types other than myeloid.

miR-146a has been previously identified as an important regulator of myeloid differentiation in the context of myelodysplastic syndrome (MDS), where it targets TRAF6 (Starczynowski et al., 2010). Since miR-146a is located on chromosome 5q, it remains to be determined whether deregulated Syk expression could also play a role in the pathogenesis of MDS with 5q deletion.

Interestingly, miR-146a has also been described as an important regulator of monocytic/macrophage development downstream of the myeloid transcription factor PU.1 (Ghani et al., 2011). This observation links miR-146a to myeloid differentiation, which is dysregulated in leukemic transformation. In our model system, overexpression of Meis1 reduces PU.1 occupancy at the putative promoter region of the miR-146a host gene. In addition, a global downregulation of PU.1 target genes suggests reduced PU.1 activity genome-wide. This can be partially explained by reduced PU.1 expression in H/M cells.

PU.1 has a dual role in the development of leukemia. On the one hand, it is required for the maintenance of MLL-driven leukemias (Aikawa et al., 2015, Zhou et al., 2014). On the other hand, graded reduction of PU.1 levels, not ablation of PU.1, has been identified as a mechanism of leukemic transformation in both human and mouse model systems (Rosenbauer et al., 2004, Rosenbauer et al., 2005, Will et al., 2015). The importance of PU.1 dysregulation is further underscored by the identification of *PU.1*-inactivating mutations in human MLL-rearranged AML (Lavallee et al., 2015). Our data link Meis1 overexpression to PU.1 downregulation, suggesting that attenuated PU.1 activity might be a functionally relevant feature of Hox/Meis-driven AML.

Our results implicate Syk in Meis1-mediated leukemic transformation. Syk potently cooperates with Hoxa9 for leukemic transformation and is strikingly similar to Meis1 with regard to its leukemogenic potential. This similarity is furthermore underscored by the ability of Syk to induce a Meis1 transcriptional program in the context of Hoxa9 overexpression. Notably, Syk does not render Hoxa9-transformed cells independent of Meis1, indicating a cell-intrinsic dependency.

Meis1 enhances Syk expression through a regulatory feedback circuit. In addition, Meis1 also upregulates the Syk activator Itgb3 and downregulates the phosphatase Ptpn6 (log fold change = −0.345, Benjamini-Hochberg adjusted p = 2.88 × 10^−17^; H/M versus H cells), a known negative regulator of Syk activity. Additional signaling effectors that might contribute to Syk activation in our circuit remain to be identified.

The largely overlapping transcriptional consequences of Syk and Meis1 led us to hypothesize that Meis1-transformed leukemias would be more addicted to Syk than to other signaling proteins such as Flt3, which has previously been shown to be dispensable for Meis1-driven leukemias (Morgado et al., 2007). Our orthogonal treatment results, based on both shRNA-mediated knockdown and pharmacological inhibition of Syk, showed that Hoxa9/Meis1-transformed leukemias were clearly more sensitive to Syk inhibition than Hoxa9-transformed leukemias.

In summary, we have identified a Meis1-dependent feedback loop involving PU.1, miR-146a, and Syk that promotes cell survival and can be targeted by Syk inhibitors. Therefore, Hoxa9/Meis1-overexpressing AML is a prime candidate for exploring the therapeutic potential of Syk inhibition.

## STAR★Methods

### Key Resources Table

**Table:** 

REAGENT or RESOURCE	SOURCE	IDENTIFIER
**Antibodies**		

Mouse monoclonal anti-CD34 BV421 (581)	BD Bioscience	Cat# 562577
Rat monoclonal anti-CD19 APCH-7 (1D3)	BD Bioscience	Cat# 560245, RRID: AB_1645233
Rat monoclonal anti-CD51 PE (RMV-7)	BD Bioscience	Cat# 551187, RRID: AB_394088
APC Annexin V	BD Bioscience	Cat# 550475, RRID: AB_2034024
Rat monoclonal anti- CD11b eFluor®450 (M1/70)	eBioscience	Cat# 48-0112, RRID: AB_1582237
Armenian Hamster monoclonal anti-CD61 PE (2C9.G3)	eBioscience	Cat# 12-0611, RRID: AB_465718
Rat monoclonal anti-CD117 APC (2B8)	eBioscience	Cat# 17-1171, RRID: AB_469430
Armenian Hamster monoclonal anti-Fc epsilon receptor I alpha PE (MAR-1)	eBioscience	Cat# 12-5898, RRID: AB_466028
Rat monoclonal anti-Ly-6A/E PE-Cyanine7 (D7)	eBioscience	Cat# 25-5981, RRID: AB_469669
Rat monoclonal anti- Ly-6G PerCP-Cyanine5.5 (RB6-8C5)	eBioscience	Cat# 45-5931, RRID: AB_906247
Rabbit polyclonal anti-Histone H3	Abcam	Cat# ab1791, RRID: AB_302613
Rabbit monoclonal anti-β-Actin (D6A8)	Cell Signaling Technologies	Cat# 8457, RRID: AB_10950489
Rabbit monoclonal anti-Btk (D3H5)	Cell Signaling Technologies	Cat# 8547, RRID: AB_10950506
Rabbit polyclonal anti-phospho-Btk (Tyr223)	Cell Signaling Technologies	Cat# 5082S, RRID: AB_10561017
Rabbit monoclonal anti-phospho-Syk (Tyr525/526) (C87C1)	Cell Signaling Technologies	Cat# 2710, RRID: AB_2197222
Rabbit monoclonal anti-PU.1 (9G7)	Cell Signaling Technologies	Cat# 2258, RRID: AB_2186909
Rabbit monoclonal anti-Syk (D1I5Q)	Cell Signaling Technologies	Cat# 12358
Rabbit polyclonal anti-Tubulin	Cell Signaling Technologies	Cat# 2144, RRID: AB_2210548
Goat polyclonal anti-GFP	Abcam	Cat# ab6673, RRID: AB_305643
Rabbit polyclonal anti-Meis1	Abcam	Cat# ab19867, RRID: AB_776272
Rabbit polyclonal anti-Syk (phospho Y348) antibody	Abcam	Cat# ab52212, RRID: AB_882779
Rabbit monoclonal anti-PU.1/Spi1 [EPR3158Y]	Abcam	Cat# ab76543, RRID: AB_1524271
Rabbit monoclonal anti-Syk (D3Z1E) XP®	Cell Signaling Technologies	Cat# 13198
Rabbit polyclonal anti-HOXA9	Novus Biologicals	Cat# NBP2-32356
Rabbit IgG	Santa Cruz	Cat# sc-2027X, RRID: AB_737197
Rabbit polyclonal anti-PU.1 (T-21)	Santa Cruz	Cat# sc-352X, RRID: AB_632289
Rabbit monoclonal anti-phospho-tyrosine (P-Tyr-1000) MultiMab™	Cell Signaling Technologies	Cat# 8954

**Chemicals, Peptides, and Recombinant Proteins**		

Dimethylsulfoxid	Applichem	Cat# A3672
7-AAD	BD Bioscience	Cat# 559925
FcR Blocking Reagent, mouse	Miltenyi Biotec	Cat# 130-092-575
Giemsa	Merck Millipore	Cat# 109204
May-Grünwald solution	Merck Millipore	Cat# 101424
Recombinant Human Flt3-Ligand	Peprotech	Cat# 300-19
Recombinant Human IL-6	Peprotech	Cat# 200-06
Recombinant Murine IL-3	Peprotech	Cat# 213-13
Recombinant Murine SCF	Peprotech	Cat# 250-03
Recombinant Murine TPO	Peprotech	Cat# 315-14
QIAzol Lysis Reagent	Qiagen	Cat# 79306
Roti®-Histofix 4 %	Roth	Cat# P087.1
PRT062607	Selleckchem	Cat# S8032
R788	Selleckchem	Cat# S2625
R406	Selleckchem	Cat# S2194
4-Hydroxytamoxifen	Sigma-Aldrich	Cat# H6278
Doxycycline hyclate	Sigma-Aldrich	Cat# D9891
G 418 disulfate salt solution	Sigma-Aldrich	Cat# G8168
Histopaque®	Sigma-Aldrich	Cat# 10831
RetroNectin® Recombinant Human Fibronectin Fragment	TaKaRa Bio Inc.	Cat# T100B
Fast SYBR® Green Master Mix	Thermo Fisher Scientific	Cat# 4385612
Pierce™ 16% Formaldehyde (w/v), Methanol-free	Thermo Fisher Scientific	Cat# 28906

**Critical Commercial Assays**		

ChIP-IT® Express	Active Motif	Cat# 53008
GeneChip® miRNA 3.0 Array	Affymetrix	Cat# 902017
Cell Proliferation Kit XTT	Applichem	Cat# A8088,1000
APC BrdU Flow Kit	BD Bioscience	Cat# 552598
PE Annexin V Apoptosis Detection Kit I	BD Bioscience	Cat# 559763
IPure kit v2	Diagenode	Cat# C03010015
CD34 MultiSort Kit, human	Miltenyi Biotec	Cat# 130-056-701
Lineage Cell Depletion Kit, mouse	Miltenyi Biotec	Cat# 130-090-858
NEBNext® Ultra™ RNA Library Prep Kit for Illumina®	New England Biolabs	Cat# E7530S/L
Dual-Luciferase® Reporter Assay System	Promega	Cat# E1910
miRNeasy Mini Kit	Qiagen	Cat# 217004
RNeasy Mini Kit	Qiagen	Cat# 74106
NE-PER™ Nuclear and Cytoplasmic Extraction Reagents	Thermo Fisher Scientific	Cat# 78833
Pierce™ BCA Protein Assay Kit	Thermo Fisher Scientific	Cat# 23227
RevertAid H Minus First Strand cDNA Synthesis Kit	Thermo Fisher Scientific	Cat# K1632
SuperSignal™ West Femto Maximum Sensitivity Substrate	Thermo Fisher Scientific	Cat# 34096
TaqMan® MicroRNA Reverse Transcription Kit	Thermo Fisher Scientific	Cat# 4366596
TaqMan® Universal Master Mix II, with UNG	Thermo Fisher Scientific	Cat# 4440038
TURBO DNA-free™ Kit	Thermo Fisher Scientific	Cat# AM1907
TaqMan® MicroRNA Assay hsa-miR-146a	Thermo Fisher Scientific	Cat# 4427975 Assay ID: 000468
TaqMan® MicroRNA Assay Gapdh	Thermo Fisher Scientific	Cat# 4331182 Assay ID: Mm99999915_g1
TaqMan® MicroRNA Assay Meis1	Thermo Fisher Scientific	Cat# 4331182 Assay ID: Mm00487664_m1
TaqMan® MicroRNA Assay Spi1	Thermo Fisher Scientific	Cat# 4331182 Assay ID: Mm00488140_m1
TaqMan® MicroRNA Assay snoRNA202	Thermo Fisher Scientific	Cat# 4427975 Assay ID: 001232

**Deposited Data**		

Mass spectrometry dataset	This paper	PRIDE Archive (PRIDE: PXD004192)
RNA-seq dataset	This paper	Short Read Archive (SRA: PRJNA322136)
miRNA microarray dataset	This paper	NCBI Gene Expression Omnibus (GEO: GSE74566)

**Experimental Models: Cell Lines**		

GP+E-86 (ATCC® CRL-9642™)	ATCC	Cat# CRL-9642
Platinum-E (Plat-E) Retroviral Packaging Cell Line	Cell Biolabs, Inc.	Cat# RV-101
293T	DSMZ	Cat# ACC 635
KG1	DSMZ	Cat# ACC 14
MV4-11	DSMZ	Cat# ACC 102

**Experimental Models: Organisms/Strains**		

B6.Cg-Mir146tm1.1Bal/J	Jackson Laboratory	Cat# 016239
B6;129-Gt(ROSA) 26Sortm1(Cre/ERT)Nat/Meis1tmloxP/ tmloxP	Miller et al., 2016	N/A
C57BL/6J	Jackson Laboratory	Cat# 000664
NOD.Cg-Prkdcscid Il2rgtm1Wjl/SzJ	Jackson Laboratory	Cat# 005557

**Oligonucleotides**		

Pre-miR miRNA Precursor hsa-miR-146a-5p	Thermo Fisher Scientific	Cat# AM17100 Asssay ID: PM10722
Pre-miR miRNA Precursor Negative Control #1	Thermo Fisher Scientific	Cat# AM17110
shGL2 TGCTGTTGACAGTGAGCGCTGGCCTTATCTGCCTCCTTAATAGTGAAGCCACAGATGTATTAAGGAGGCAGATAAGGCCATTGCCTACTGCCTCGGA	Sigma-Aldrich	N/A
shSyk-1 TGCTGTTGACAGTGAGCGCTGGCCTTATCTGCCTCCTTAATAGTGAAGCCACAGATGTATTAAGGAGGCAGATAAGGCCATTGCCTACTGCCTCGGA	Sigma-Aldrich	N/A
shSyk-2 TGCTGTTGACAGTGAGCGATGGAATAATCTCAAGGATCAATAGTGAAGCCACAGATGTATTGATCCTTGAGATTATTCCACTGCCTACTGCCTCGGA	Sigma-Aldrich	N/A
hHOXA9 fwd AAAACAATGCTGAGAATGAGAGC	Sigma-Aldrich	N/A
hHOXA9 rev TATAGGGGCACCGCTTTTT	Sigma-Aldrich	N/A
hMEIS1 fwd GCATGAATATGGGCATGGA	Sigma-Aldrich	N/A
hMEIS1 rev CATACTCCCCTGGCATACTTTG	Sigma-Aldrich	N/A
hACTB fwd TCCCTGGAGAAGAGCTACG	Sigma-Aldrich	N/A
hACTB rev GTAGTTTCGTGGATGCCACA	Sigma-Aldrich	N/A
miR146a (1) fwd CCACCTTAAAGCCAGCAGAG	Sigma-Aldrich	N/A
miR146a(1) rev CCTGACCAGCACTTCCTCAG	Sigma-Aldrich	N/A
PU.1 -13.7 fwd AGGCAGAGCACACATGCTTC	Sigma-Aldrich	N/A
PU.1 -13.7 rev CTTCTGGGCAGGGTCAGAGT	Sigma-Aldrich	N/A
FLT3 exon9 fwd TTTGCACTCGTAGCAAATGG	Sigma-Aldrich	N/A
FLT3 exon9 rev GTTCAGCTGCCAAAGAGAGG	Sigma-Aldrich	N/A
Exon 7 CTL fwd TTGGAATAGAGACCATGATGACAC	Sigma-Aldrich	N/A
Exon 7 CTL rev GTTATCCCCACTGTGTGAAGTATG	Sigma-Aldrich	N/A
NDF fwd AGCTTCATTTGAAGTTCCCTATTG	Sigma-Aldrich	N/A
NDF rev TATTAGGTGGATCCAAGCTTCATT	Sigma-Aldrich	N/A
DSA7/8 s CACCGAGGAACTGTTCACCGCGGCG	Sigma-Aldrich	N/A
DSA7/8 as AAACCGCCGCGGTGAACAGTTCCTC	Sigma-Aldrich	N/A
mmu-mir146a/b s CACCGTCTGAGAACTGAATTCCAT	Sigma-Aldrich	N/A
mmu-mir146a/b as AAACATGGAATTCAGTTCTCAGAC	Sigma-Aldrich	N/A
NTC s CACCGTTCCGGGCTAACAAGTCCT	Sigma-Aldrich	N/A
NTC as AAACAGGACTTGTTAGCCCGGAAC	Sigma-Aldrich	N/A
Itgb3 s CACCGATTGAGTTCCCAGTCAGTG	Sigma-Aldrich	N/A
Itgb3 as AAACCACTGACTGGGAACTCAATC	Sigma-Aldrich	N/A

**Recombinant DNA**		

pMD2.G	addgene	Cat# 12259
psPAX2	addgene	Cat# 12260
psiCHECK™-2 Vector	Promega	Cat# C8021
LT3-GEPIR	Fellmann et al., 2013	N/A
MSCV-Hoxa9-PGK-neo	Kroon et al., 1998	N/A
MSCV-Meis1-IRES-YFP	Pineault et al., 2003	N/A
pLentiCRISPRv2	Sanjana et al., 2014	N/A

**Software and Algorithms**		

BioConductor v3.2	Huber et al., 2015	http://www.bioconductor.org
Bowtie2 v2.2.3	Langmead and Salzberg, 2012	http://bowtie-bio.sourceforge.net/bowtie2
DESeq2 v1.10.1	Love et al., 2014	http://www.bioconductor.org/packages/release/bioc/html/DESeq2.html.
Gencode annotation vM7	Gencode	https://www.gencodegenes.org/mouse_releases/7.html
FastQC v0.11.4	Babraham Bioinformatics	http://www.bioinformatics.babraham.ac.uk/projects/fastqc/
GeneSpring 13.1	N/A	http://www.genomics.agilent.com/article.jsp?pageId=2141
ISMARA client v1.0.1	Balwierz et al., 2014	https://ismara.unibas.ch/ISMARA/client/
MACS2 v2.1.0	Zhang et al., 2008	https://github.com/taoliu/MACS
MaxQuant version v1.5.2.8	Cox and Mann, 2008	http://www.coxdocs.org/doku.php?id=maxquant:common:download_and_installation
Perseus version v1.5.2.4	N/A	http://www.coxdocs.org/doku.php?id=perseus:common:download_and_installation
R v3.2.3	R Core Team, 2016	https://www.r-project.org
STAR v2.4.2a	Dobin et al., 2012	https://github.com/alexdobin/STAR
UniProt human database	UniProt	http://www.uniprot.org/

**Other**		

L-ARGININE:HCL (U-13C6, 99%; U-15N4, 99%)	Euriso-top	Cat# CNLM-539
L-Lysine:2HCl, “(13C6, 99%; 15N2, 99%)”	Euriso-top	Cat# CNLM-291
Publicly available data (Meis1 ChIP-seq)	Gene Expression Omnibus (GEO)	Cat# GSM842248, Cat# GSM842251
Titansphere 10μm, 500mg	GL Science	Cat# 5020-75010
LS Columns	Miltenyi Biotec	Cat# 130-042-401
C18 stage-tip	Rappsilber et al., 2003	N/A
L-Prolin	Roth	Cat# 1713.2
dialyzed FCS	Sigma-Aldrich	Cat# F0392
L-Arginine	Sigma-Aldrich	Cat# A5006
L-Lysine dihydrochloride	Sigma-Aldrich	Cat# L5751
MethoCult™ GF M3534	Stemcell Technologies	Cat# 03534
StemSpan™ SFEM	Stemcell Technologies	Cat# 09600, 09650
DMEM for SILAC	Thermo Fisher Scientific	Cat# 88420

### Contact for Reagent and Resource Sharing

Further information and requests for resources and reagents should be directed to and will be fulfilled by the Lead Contact, Thomas Oellerich (thomas.oellerich@kgu.de).

### Experimental Model and Subject Details

#### Mice

All animal experiments were performed according to the regulations of the United Kingdom Home Office and German authorities. *Meis1*^*tmloxP/tmloxP*^ mice crossed to B6;129-*Gt(ROSA)26Sor*^*tm1(Cre/ERT)Nat*^/J mice were obtained as previously described (Miller et al., 2016) and maintained at the British Columbia Cancer Agency Animal Resource Centre (ARC) with all protocols approved by the University of British Columbia Animal Care Committee (Certificate A13-0063). C57BL/6J mice for transplantation experiments were obtained from Jackson Laboratory (Bar Harbor) and maintained at the Zentrale Forschungseinrichtung (ZFE) of the Goethe University of Frankfurt. Donor (8-10 weeks) and recipient mice (10-12 weeks) were reared in groups of up to 5 mice per cage. Mice from transplantation assays were sacrificed after visible characteristics of AML. B6/miR-146a^-/-^ mice (8-10 weeks) (Boldin et al., 2011) were purchased from the Jackson Laboratory (Bar Harbor). All mice were female with a weight >20g.

#### Cell Lines

293T (DSMZ), the ecotropic GP+E86 (ATCC) and Platinum-E (PlatE, Cell Biolabs) packaging cell lines were cultured in DMEM (Life Technologies) with 10% heat-inactivated (h.i.) FCS (Sigma-Aldrich), 2 mM L-Glutamine (Lonza), 100 U/ml Penicillin and 100 μg/ml Streptomycin (Life Technologies).

#### Primary Cells

Murine bone marrow cells were isolated by flushing femur and tibia of the hind legs of two to three mice. Bone marrow cells from individual animals were pooled for further processing. Mouse bone marrow cells were cultured in DMEM with 10% h.i. FCS (Sigma-Aldrich), 2 mM L-Glutamine (Lonza), 100 U/ml Penicillin and 100 μg/ml Streptomycin (Life Technologies) supplemented with 10ng/ml murine recombinant IL3, 10 ng/ml human recombinant IL6, and 100ng/ml murine recombinant SCF (all Peprotech) (Argiropoulos et al., 2008).

CD34+ mononuclear cells were isolated from human bone marrow aspirates. Bone marrow aspiration from healthy donors was performed at the Institute of Transfusion Medicine and Immunohematology of Goethe University and German Red Cross Blood Donor Service in Frankfurt. Use of the bone marrow aspirates for research purposes was approved by the Ethics Committee of the University of Frankfurt (329-10) and donors gave written consent for use of the samples. Short term culture of CD34+ bone marrow cells from healthy donors was done in StemSpan™ Serum-Free Expansion Medium (SFEM, Stemcell Technologies) supplemented with 100 ng/ml murine SCF, 100 ng/ml murine TPO, 100 ng/ml human FLT3-L and 100 ng/ml human IL6 (all Peprotech).

#### Human Bone Marrow Biopsies

Bone marrow biopsies from 115 AML patients treated at the University Hospital Frankfurt, Germany, were obtained for histological staining from the biobank of the local University Cancer Center. Use of the biopsies for research purposes was approved by the Ethics Committee of the University of Frankfurt (SHO-04-2014). All patients gave written consent for use of their samples. Patient characteristics are summarized in Table S2.

### Method Details

#### Retroviral Infection of Lineage-Depleted Bone-Marrow Cells

Bone marrow cells were harvested from C57BL/6J or conditional Meis1 knockout mice, and lineage-negative cells were obtained by negative selection using a Lineage Cell Depletion Kit (mouse) (Miltenyi Biotec) following the manufacturer's instructions. 3-5x10^5^ lineage-negative cells were retrovirally infected with Hoxa9 alone or Hoxa9 and Meis1 by co-culture with 1x10^6^ GP+E86 cells containing MSCV-Hoxa9-PGK-neo (for 3 days) or MSCV-Meis1-IRES-YFP (for 1 day) in 2 ml medium in a 6-well plate in the presence of 10 μg/ml polybrene (Sigma-Aldrich). Hoxa9 cells were selected with 0.6 mg/ml G418 (Sigma-Aldrich) for at least 5 days. After selection, cells were sorted with a FACS BD Aria III cell sorter. Meis1 knock-out in Meis1^fl/fl^ mice was induced by treatment with 4-hydroxy-tamoxifen (4-OHT) (Sigma-Aldrich) at a final concentration of 100 nM.

#### Lentivirus Production and Lentiviral Transduction

VSV-G pseudotyped virus particles of CRISPR (pLentiCRISPRv2-BFP) or shRNA vectors (LT3-BEPIR) were produced by transient transfection of subconfluent HEK293T cells with the packaging Plasmid psPAX2 (Addgene plasmid #12260) and the envelope plasmid pMD2.G (Addgene plasmid #12259, both were a gift from Didier Trono) using calcium phosphate or polyethylenimine (Bender et al., 2016). Ecotropic retrovirus for hSYK in the pRRL.PPT.SFFV.IRES.eGFP.wPRE vector was generated using the Platinum-E (PlatE) packaging cell line (Cell Biolabs). After 36 and 60 hours, cell culture supernatants were collected, filtered (0.45 μm) and viral particles were spun (1243xg, 30 min, 32°C) onto 24-well cell culture plates which had been coated with RetroNectin (Takara Bio USA Inc) before according to the manufacturer's instructions. Finally, target cells were seeded and spun (200xg, 5 min, 32°C) onto the virus-coated cell culture plate. To increase transduction efficiency of VSV-G pseudotyped lentivirus, cells were additionally treated with concentrated virus which was enriched by ultracentrifugation (51610xg, 2 hours, 4°C) in an OPTIMA™ XPN-80 ultracentrifuge using the SW32 Ti rotor (Beckman Coulter GmbH). Transduced cells were selected with puromycin or fluorescence activated cell sorting. shRNA expression in transduced cells was induced by addition of 1 μg/ml doxycyclin (Sigma-Aldrich) to the cell culture medium. Of the hSYK transduced cells, cells with an approximately 3-fold increase of SYK expression were sorted by FACS.

#### XTT Viability/Proliferation Assay

Proliferation was assessed by XTT (sodium 2,3,-bis(2-methoxy-4-nitro-5-sulfophenyl)-5-[(phenylamino)-carbonyl]-2H-tetrazolium) inner salt) assay: 5000 cells/well were seeded in regular growth medium in quadruplicate into 96-well plates. After seeding (day 0) and on days 2 and 3, XTT reagent (Applichem) was added and absorption was measured after 4 h by using a Tecan Infinite 200Pro plate reader. Medium alone was used as blank.

#### Colony Formation Assay

Colony-forming cells (CFCs) were assayed in methylcellulose (Methocult M3534; StemCell Technologies Inc.) as described previously (Heuser et al., 2007). 100 cells were mixed with 1 ml methylcellulose and inhibitor or DMSO as control. Cells were incubated at 37°C under 5% (v/v) CO_2_. Colonies were evaluated microscopically 7 days after plating by using standard criteria. For replating, cells were eluted from the methylcellulose, washed, counted and replated as described above.

#### Transplantation, Monitoring and Analysis of Animals

7.5x10^4^ cells were transplanted together with 2x10^5^ support cells by injection into the tail vein of lethally irradiated (9.5 Gy) recipient mice (C57BL/6J). Support cells were isolated from C57BL/6J mice and purified on a Ficoll gradient (Sigma-Aldrich,).

After transplantation with the inducible shRNA-based Syk knock-down cells, mice were treated with doxycyclin as described in (Zuber et al., 2011). The R788 treatment was performed as described in (Hahn et al., 2009).

Blood, spleen, liver and bone marrow were isolated from mice for further analysis. Blood counts were analyzed with ScilVet abc animal blood cell counter (Scil Animal Care Company). Cells from spleen and bone marrow were incubated for 10 min with erythrocyte lysis buffer (155 mM NH_4_Cl, 10 mM KHCO_3_, 0.1 mM EDTA) and then washed twice with 2% FCS in PBS. Staining was performed with antibodies against the following cell surface markers: Gr-1, Mac-1, c-Kit, Sca-1 and CD19. 7-AAD was used as viability dye.

Blood smears and purified cells from bone marrow and spleen were centrifuged on object slides (2x10^5^ cells/slide). Subsequently, cells were fixed for 10 min with methanol and stained with a May-Grünwald solution for 8 min followed by Giemsa (both Merck Millipore) staining for 20 min.

Parts of spleen and liver were fixed and stored in Roti®-Histofix 4% (Roth) and subjected to routine HE staining and IHC for GFP/YFP at the Histology-Core Facility of the Georg-Speyer-Haus, Frankfurt.

Primary human AML samples derived from BM MNCs were depleted of CD3+ T lymphocytes and transplanted via tail vein injection into 6-week-old NOD–Scid *Il2rg*null (NSG) mice (Jackson Laboratory) conditioned with 200 cGy of gamma irradiation.

#### Isolation of CD34+ Mononuclear Cells from Human Bone Marrow Aspirates

Isolation of bone marrow mononuclear cells (BM-MNCs) from human bone marrow aspirates was achieved by Ficoll density centrifugation (400xg, RT, 45 min). CD34+ BM-MNCs were isolated using the human CD34 MultiSort Kit (Miltenyi Biotec) following the manufacturer's instructions. Briefly, BM-MNCs were washed twice with MACS buffer (PBS containing 0.5% bovine serum albumin (BSA) and 2 mM EDTA), passed through a nylon mesh to remove cell clumps and incubated in MACS buffer supplemented with FcR Blocking Reagent and CD34 MultiSort MicroBeads for 30 min at 4°C. Excess MicroBeads were washed off and magnetic separation was done manually using LS columns. After elution, cells were washed once in MACS buffer and resuspended in SFEM supplemented with diverse cytokines as described above. Purity of the isolated cells was verified by flow cytometry. For apoptosis measurements, 2x10^4^ cells were seeded in 100 μl culture medium and treated with DMSO (control), 1 μM, 5 μM and 10 μM R406 or PRT062607 for 24 hours. Annexin V staining was done as described below.

#### Flow Cytometry

For flow-cytometry, 2x10^5^ cells were washed twice with 2% FCS in PBS and stained for 20 min at 4°C in the dark with the following antibodies: PerCP-Cy5.5-conjugated anti-mouse Gr1, V450-conjugated anti-mouse Mac1, APC-conjugated anti-mouse c-kit, PE-Cy7-conjugated anti-mouse Sca1, PE-conjugated anti-mouse FcɛRI, PE-conjugated anti-mouse/rat CD61 (Itgb3, all from eBioscience), PE-conjugated anti-mouse CD51, BV421-conjugated anti-human CD34 and APC-H7-conjugated anti-mouse CD19 (both BD Bioscience). Excess antibody was removed by washing the cells at least twice with 2% FCS in PBS. 7-AAD (BD-Bioscience) was used for dead-cell exclusion.

#### Generation of CRISPR Constructs

pLentiCRISPRv2 vectors (Sanjana et al., 2014) containing the different sgRNAs were obtained by target-specific oligonucleotide annealing using the GoldenGate protocol. Oligonucleotide names and sequences (5’-3’) are listed in the key resource table. The puromycin resistance cassette in pLentiCRISPRv2 was replaced by blue fluorescent protein (BFP) using standard cloning techniques to allow fluorescence activated cell sorting of transduced cells.

#### Generation of shRNA Constructs

Doxycycline-inducible lentiviral shRNA vectors were provided by Johannes Zuber (Research Institute of Molecular Pathology, Vienna, Austria) (Fellmann et al., 2013). PU.1 knockdown was performed as described (Zhou et al., 2014). For the knockdown of Syk in mouse BM cells, the GFP expression cassette under the Dox-inducible T3G promoter of the LT3-GEPIR vector was exchanged to BFP by overlap PCR. The resulting vector was named LT3-BEPIR. The 97nt template hairpin oligos for shRNA cloning were designed with the RNAi Central shRNA retriever tool (http://cancan.cshl.edu/RNAi_central/RNAi.cgi?type=shRNA) by providing the accession number of mouse Syk, transcript variant 1 (NM_011518). The synthetic hairpin oligos served as templates for PCR amplification using the primer sequences for de novo generation of miR-E shRNAs (Fellmann et al., 2013). Finally, the shRNAs were cloned into the LT3-BEPIR vector via the XhoI and EcoRI restriction sites. An LT3-BEPIR vector containing the non-targeting GL2 shRNA against the *Renilla* Luciferase gene in the pGL2-basic cloning vector (GenBank X65323.2) was used as control. The sequences of the template hairpin oligos used for cloning are listed below.

#### Immunohistochemistry

Tumor tissues were fixed and stained as described (Oellerich et al., 2015). Briefly, biopsy samples for histological stains were sliced and stained with antibodies against HOXA9 (Novus Biologicals; dilution 1:500), MEIS1 (Abcam; dilution 1:2500), PU.1/Spi1 (Abcam; dilution 1:200), SYK (Cell Signaling Technologies; dilution 1:100) and pY348-SYK (Abcam; dilution 1:100). Two independent pathologists performed evaluation of all tissue samples based on a three-level staining score (0 = negative; 1 = weakly positive; 2 = positive). Overexpression of HOXA9, MEIS1, PU.1 and (p)SYK was defined by a score of 2.

#### Cell Cycle Analysis

Cell cycle analysis was performed using the BD Pharmingen APC BrdU Flow Kit (BD Bioscience) according to the manufacturer’s protocol.

Briefly, 1x10^6^ transduced mouse BM cells were cultured for 48h prior to labeling with 10 μM BrdU for 1 hour. Cells were harvested, washed once with Staining Buffer (1x PBS supplemented with 3% h.i. FCS and 0.09% sodium azide) and incubated with Cytofix/Cytoperm buffer for 15 min on ice. After washing with PermWash buffer, cells were permeabilized with Cytoperm Plus buffer for 10 min on ice, washed with PermWash buffer, incubated again in Cytofix/Cytoperm buffer for 5 min on ice followed by another PermWash buffer washing step. BrdU epitopes were then exposed by treating the cells with 0.4 mg/ml DNase I for 1 hour at 37°C. After removal of the DNase by washing the cells with PermWash buffer, they were incubated with 1 μl APC-conjugated BrdU antibody in 50 μl PermWash buffer for 20 min at RT in the dark. Subsequently, cells were subjected to a final PermWash washing step, resuspended in 20 μl 7-AAD and incubated for 5 min at RT in the dark. After addition of 1 ml Staining Buffer, cells were analysed using a BD LSRFortessa flow cytometer.

#### Annexin V Staining

For detection of apoptotic cell death, the Annexin V Apoptosis Detection Kit I (BD Bioscience) was used according to the manufacturer’s instructions. Briefly, cells were harvested and washed once with PBS, resuspended in 200 μl 1x Annexin V binding buffer containing 5 μl Annexin-PE or Annexin-APC and incubated for 15 min at RT in the dark. After one washing with 1x Annexin binding buffer, cells were resuspended in 1x Annexin V binding buffer containing 5 μl 7-AAD and incubated for 10 min at RT in the dark. Finally, 200 μl 1x Annexin V binding buffer were added and cells were analysed using a BD LSRFortessa flow cytometer.

#### RNA Isolation and Reverse Transcription

Cells were collected by centrifugation, washed once with PBS and lysed in Qiazol (Qiagen). RNA isolation was performed according to the TRIzol reagent (Life Technologies) protocol. For gene expression analysis, RNA was treated with DNase to eliminate contaminating DNA using the Turbo DNA-free kit according to the manufacturer's protocol (Ambion). For analyses of gene and pri-miR expression, 250 ng RNA were reverse transcribed into cDNA using 5 μM Random Hexamers, 20 U RiboLock RNase inhibitor, 1mM of each dNTP and 200U RevertAid H Minus M-MuLV reverse transcriptase in a PCR machine (25°C for 5 min, 42°C for 60 min, 70°C for 5 min) according to the RevertAid H Minus First Strand cDNA Synthesis Kit manual (Thermo Fisher Scientific). cDNA synthesis for mature miRNA detection was done according to the TaqMan MicroRNA Assay protocol (Applied Biosystems).

#### RNA Sequencing and Data Analysis

RNA was extracted from five replicates (∼10^7^ cells each) for each cell type (H, H/M and H/S) using the RNeasy Mini Kit (Qiagen) with on-column DNase I digestion. RNA quality and quantity were assessed using an Agilent Bioanalyzer 2100 and RNA Nano Chips (Agilent Technologies). RNA integrity numbers ranged between 9.7 and 10. Poly-A enrichment and preparation of sequencing libraries was conducted with 1 μg total RNA from each sample using the NEB Next Ultra RNA Library Preparation Kit (NEB). Single-end sequencing (75 bp) was conducted on a NextSeq500 (Illumina) following standard procedures. The average read counts per group were: H: 22.5M (SD: 1.2M); H/M: 22.1M (SD: 0.3M), H/S: 23.2M (SD:1.4M). Read alignment to the mouse genome reference sequence GRCm38/mm10 was performed using STAR v2.4.2a (Dobin et al., 2012). Differential gene expression analysis was conducted using DESeq2 v1.10.1 (Love et al., 2014).

#### Immunoblotting

Lysis of cells was performed with NP40-containing lysis buffer (150 mM NaCl, 50 mM Tris at pH 7.5–7.8, 5 mM NaF, 0.5% NP40, 1 mM sodium vanadate, Complete protease inhibitor cocktail (Roche) for 10 min on ice followed by centrifugation to remove cell debris. For nuclear proteins, NE-PER™ Nuclear and Cytoplasmic Extraction Reagents (Thermo Fisher Scientific) were used according to the manufacturer’s protocol. Protein concentrations were determined with the Pierce™ BCA Protein Assay Kit (Thermo Fisher Scientific). Proteins were separated on precast 4–15% Mini Protean TGX gels at 190 V using the Mini Protean electrophoresis system and TGS buffer and were blotted onto nitrocellulose membranes at 70V for 2 h in a Mini Trans-Blot Cell by using the TG buffer supplemented with 20% methanol (all BioRad). All antibodies were used as recommended by the manufacturer.

#### Dual luciferase Assay

Synthetic oligonucleotides containing the two miR-146a binding sites from the mouse Syk 3′-UTR or mutated versions thereof in duplicate, all ending with XhoI and NotI restriction sites, were annealed and cloned into the PsiCheck-2 dual luciferase vector (Promega). Additionally, a dual luciferase vector comprising the complete mouse Syk 3′-UTR was used (Genecopoeia). For the dual luciferase assays, HEK293T cells were seeded in 24-well plates and co-transfected with 0.2 ng dual luciferase vector and 20 pmol Pre-miR control or miR-146a precursor (Ambion) using Lipofectamine 2000 (Invitrogen). 48h after transfection, cells were lysed and firefly and renilla luciferase activities were measured with the Dual-Luciferase-Assay System (Promega) according to the manufacturer's protocol on a Tecan Infinite 200Pro plate reader.

#### miRNA Expression Profiling

Expression analysis of miRNAs was performed using an Affymetrix GeneChip miRNA 3.0 array. miRNAs were isolated with the miRNeasy Kit (Qiagen). Data analysis was performed using GeneSpring 13.1. Raw data were processed with the RMA algorithm and normalized by quantile normalization. miRNAs with expression values below the 20th percentile were removed. A difference in expression between Hoxa9 and Hoxa9/Meis1 cells was considered statistically significant if q value <0.01 (moderated t-test and Benjamini-Hochberg multiple testing correction) and absolute log2 fold change >1.

#### Chromatin Immunoprecipitation

ChIP was performed by using the ChIP-IT^®^ Express Kit (Active Motif) according to the manufacturer's instructions. Briefly, 2x10^7^ H and H/M cells, respectively, were fixed with methanol-free formaldehyde (Life Technologies) at a final concentration of 1% for 10 min. After neutralization with glycine, cells were lysed in lysis buffer with protease inhibitors and nuclei were prepared according to the manufacturer's instructions (Covaris). Samples were sonicated to an average DNA length of 200–400 bp with a M220 Focused-ultrasonicator™ (Covaris). ChIP was carried out using 5 μg of anti-PU.1 antibody (Santa Cruz) or rabbit IgG (Santa Cruz), respectively. DNA was purified with IPure kit (Diagenode) and target regions were assessed by qPCR.

#### Quantitative PCR Analysis

Mouse *Syk*, *Spi1/PU.1* and *Meis1* were detected with commercial TaqMan Gene Expression assays (Applied Biosystems). GAPDH was used for normalization. Human *HOXA9* and *MEIS1* expression was measured with SYBR Green and normalized to α-actin using the primers listed in the Key Resources Table. qPCR expression data were normalized using the ΔΔCT method (Livak and Schmittgen, 2001).

miRNA expression was measured using TaqMan MicroRNA Assays designed for mmu-miR-146a (Applied Biosystems) according to the manusfacturer's protocol. Sno202 served as endogenous control. For detection of miRNA primary transcripts (Pri-miR), a commercial TaqMan Gene Expression Assays for mmu-miR-146a (Applied Biosystems) was used and GAPDH served as endogenous control.

Quantitative PCR for confirmation and quantitation of Meis1 knock-out in Meis1^fl/fl^ mice was performed as previously described (Miller et al., 2016) using a primer-set NDF for detection of non-deleted floxed *Meis1* and a control primer-set detecting a region in exon 7 of *Meis1.* PCR was performed using SYBR Green (Applied Biosystems) and analyzed using the ΔΔCT method.

Quantitative PCR for the DNA from chromatin immunoprecipitation was performed using SYBR Green (Bio-Rad) for a region -10kb upstream of miR-146a, a positive control locus at -13.7kb upstream of the PU.1 transcription start site and a negative control region in exon9 of FLT3. PU.1 and IgG binding were normalized to input control using the ΔΔCT method (Livak and Schmittgen, 2001). The primer sequences are listed in the Key Resources Table.

#### Time-lapse Microscopy and cell Tracking

Time-lapse microscopy and tracking of H and H/M cells in the presence or absence of the SYK inhibitor R406 (500 nM) was performed as previously described (Haetscher et al., 2015, Rieger et al., 2009) until the fate of the cell (death or division) was determined. The time point of death was calculated from the beginning of the movie until the cell died. Dead cells were depicted by their shrunken, non-refracting and immobile appearance. The cell death proportion is calculated based on the number of cells in each generation that undergo either division or death. Cell tracking was carried out manually.

#### SILAC Labeling

SILAC-labeling was performed with SILAC DMEM (Thermo Fisher Scientific) supplemented with 10% h.i. dialyzed FCS (Sigma-Aldrich), 100 U/ml Penicillin, 100 μg/ml Streptomycin (Life Technologies) and heavy amino acid isotopes (^13^C_6_^15^N_4_L-arginine and ^13^C_6_^15^N_2_L-Lysine) (Cambridge Isotope Laboratories) or regular (light) amino acids (^12^C_6_^14^N_4_L-arginine and ^12^C_6_^14^N_2_L-Lysine, Sigma-Aldrich).

#### Phospho-peptide Enrichment and Mass Spectrometry

Antibody-based enrichment for tyrosine-phosphorylated peptides (pYome) was performed using the PTMScan Phospho-Tyrosine rabbit mAB (P-Tyr-1000) Kit as described in the manufacturer’s instructions (Cell Signaling Technology) and according to Rush *et al* (Rush et al., 2005).

Briefly, SILAC-labeled protein lysates were mixed in equimolar amounts and proteins were reduced with DTT and alkylated with IAA. The urea concentration was lowered to 2 M with 20 mM HEPES before overnight digestions with trypsin. Resulting peptides were purified with Sep-Pak tC18 cartridges and lyophilized. Immunoprecipitation was performed with the anti-phosphotyrosine-specific antibody P-Tyr-1000 (Cell Signaling Technology). Peptides were eluted under acidic conditions from the beads and purified with STAGE-Tips.

For the enrichment of the global phosphoproteome (GPome), equimolar mixed, SILAC-labeled proteins were precipitated with acetone. The pellet was dissolved in 1% RapiGest Surfactant (w/v). Proteins were reduced at a final concentration of 10 mM DTT for 1 h at 65°C and reduced with CAA at a final concentration of 20 mM for 1 h at 37°C. Proteins were digested with trypsin in the presence of 0.1% RapiGest at 37°C overnight. For RapiGest degradation the digest was acidified to 1% TFA and incubated for 2 h at 37°C. RapiGest precipitations were cleared by centrifugation with max. rpm at RT. The peptide containing supernatant was dried in a SpeedVac concentrator. Peptides were fractionated by strong cation exchange (SCX) as described by Gruhler et al. with some modifications (Gruhler et al., 2005).

Briefly, SCX chromatography was performed on a FPLC system (SMART, Pharmacia) using an ammonium formate based buffer system. Buffer A had a final concentration of 10 mM ammonium formate and buffer B had a final concentration of 500 mM ammonium formate both at pH 2.7 and containing 30% ACN (v/v). 20 Fractions were collected over a 50 min gradient with a flow rate of 100 μL/min. The first twelve fractions were dried in a SpeedVac concentrator and subsequently used for phospho-peptide enrichment or stored at -20°C until use. The following TiO_2_ spin column phosphopeptide enrichment was performed with minor modifications as described by Larsen et al. (Larsen et al., 2005). Dried peptides were suspended by intensive vortexing and sonication in 60 μL of loading solution. All following centrifugation steps were performed with 3000 rpm at RT for 5 min. In-house made titanium dioxide spin columns were equilibrated with 60 μL of loading solution followed by sample loading. Phosphopeptides bound to TiO_2_ beads were washed three times with 60 μL loading solution and five times with 60 μL washing solution. Phosphopeptides were eluted with a 0.6 N solution of NH_4_OH (pH ≥ 10.5), dried in a SpeedVac concentrator and stored at -20°C until mass spectrometric analysis.

Phosphorylated peptides were analyzed on an EASY n-LC 1000 (Thermo Scientific) coupled to a hybrid quadrupole-Orbitrap mass spectrometer (Q Exactive, Thermo Scientific). Peptides were separated on an analytical column (75 μm x 200 mm, ReproSil-Pur 120 C18-AQ, 3 μm, Dr. Maisch GmbH, packed in-house) using a 90 min (GPome) or 120 min (pYome) linear gradient, respectively. The Q Exactive was operated in a DDA selecting the twelve most abundant precursors for HCD fragmentation in the collision cell with an isolation window of 2 *m/z* and a NCE of 28.

Raw files were processed with MaxQuant v1.5.0.25 against the UniProtKB/Swiss-Prot mouse database (downloaded July 2014). Cysteine carbamidomethylation was set as fixed modification, methionine oxidation and serine, threonine and tyrosine phosphorylation for both the pYome and the global phosphoproteome dataset as variable modification. The ‘Minimum Andromeda Score’ and ‘Delta score’ for modified peptides was set to 40 and 6, respectively. The following parameters were applied: the MS1 first search peptide tolerance was set to 20 ppm and the main search peptide tolerance to 4.5 ppm. The FTMS MS/MS tolerance was set to 20 ppm, a false discovery rate (FDR) of 1% for peptide spectrum matches (PSM), protein and site decoy was applied. The ‘Re-quantify’ option of MaxQuant was enabled. Unique, razor, unmodified, N-terminally acetylated and M oxidized peptides were used for protein quantitation and the minimum ratio count required was set to 2.

For protein expression analysis, SILAC-labeled H and H/M cell lysates were mixed in a 1:1 ratio. A total of 100 μg protein was separated by SDS-Page using pre-cast Bis-Tris minigels (NuPAGE Novex 4–12%, Life Technologies) and visualised by staining with Coomassie Brilliant Blue (Serva). Each lane was cut into 23 slices, reduced with dithiothreitol (DTT, Sigma-Aldrich) and alkylated with iodoacetamide (IAM, Sigma-Aldrich), digested in-gel with trypsin (Serva), extracted and analysed by mass spectrometry as described in (Corso et al., 2016).

### Quantification and Statistical Analysis

#### Proteome Data Analysis

The MS raw files were processed by MaxQuant (Cox and Mann, 2008) (version 1.5.2.8) and MS/MS spectra were searched against UniProt human database (downloaded on Feb, 2015; 89,796 entries) via the Andromeda search engine (Cox et al., 2011). Mass tolerance after recalibration of precursor mass and fragment ion mass were set as 6 and 20 ppm, respectively. Allowed variable modifications included protein deamidation (N), oxidation (M) and phosphorylation (STY). Cysteine carbamidomethylation was defined as a fixed modification. Minimal peptide length was set to 7 amino acids with the maximum of two enzymatic mis-cleavages. The false discovery rate (FDR) was set to 1% for both peptide and protein identifications. Intensities of all identified peptides were determined by MaxQuant with options “match between runs” and “re-quantify”.

Subsequent data analysis was conducted with Perseus (version 1.5.2.4). After removing all decoy hits and potential contaminant entries, identified phosphosites with localization probability smaller than 0.75 were filtered out. All SILAC ratios were log2 transformed and those with absolute log2 *Z* score >1 were considered significantly regulated.

#### RNA-seq data Analysis

Data quality control was performed with FastQC v0.11.4 and revealed no appreciable technical artefact. Reads were aligned to the mouse reference genome (mm10, Ensembl GRCm38 release 82) using STAR v2.4.2a (Dobin et al., 2012), clipping the first 5 nucleotides (--clip5pNbases 5). Gene count tables were generated while mapping, using Gencode vM7 annotations. All downstream analyses were carried out using R v3.2.3 (R Core Team, 2016) and BioConductor v3.2 (Huber et al., 2015). Exploratory analyses and differential gene expression analysis were carried out with DESeq2 v1.10.1 (Love et al., 2014). For sample clustering and principal component analysis, genes with zero counts across all samples were removed from the analysis. For differential expression analysis, the Wald test was used for pairwise comparisons, whereas a likelihood ratio test was used to extract significant differences across all three conditions.

#### Motif Activity Analysis

Motif activity analysis was performed using the standalone ISMARA (Integrated System for Motif Activity Response Analysis) client (Balwierz et al., 2014). For this analysis, reads were aligned to the mm9 mouse reference genome (GRCm37) using STAR as described above. ISMARA profiles were averaged by taking into account the experimental design.

#### ChIP-seq data Analysis

Meis1 ChIP-seq data from (Huang et al., 2012) were retrieved from the Short Read Archive (SRA) and aligned to the mouse reference genome (mm10) using Bowtie2 (Langmead and Salzberg, 2012) with standard parameters. Peaks were called using MACS2 v2.1.0 (Zhang et al., 2008) using a q-value threshold of 0.05. The peak union from two biological replicates was used for the analysis.

#### AML patient Survival Analysis

For AML patients time-to-event data were observed using Kaplan-Meier analysis with survival end point definitions as described in (Cheson et al., 2003). For quantitative assessment hazard ratios and 95% confidence intervals (CIs) of hazard ratios were computed based on Cox proportional hazards model. Significant survival association was assessed using the log-rank test. Time-to-event data was analyzed using the R 'survival' package (version 2.38). Statistical analyses were performed in R (version 3.2.2). A two-sided p value < 0.05 was considered significant.

#### hSyk Expression

To quantify the expression of human Syk (hSyk) in H/S cells, the sequence of the lentiviral construct containing the hSyk cDNA was used to create a Bowtie2 index. RNA-seq reads were then aligned to this index using Bowtie2, allowing no mismatch in a seed of 31nt.

### Data and Software Availability

#### Data Resources

Mass spectrometry data (PRIDE: PXD004192) have been deposited to the PRIDE Archive. RNA-seq data (SRA: PRJNA322136) have been deposited to the Short Read Archive. miRNA microarray data (GEO: GSE74566) have been deposited to the NCBI Gene Expression Omnibus.

#### Software

Software programs used in this study were from publicly available resources. Please refer to Key Resources Table for more details.

## Author Contributions

T.O. conceived the study together with T. Berg, F.C., and K.H.; S.M. and C.D. performed the majority of experiments; F.C. performed computational analyses; S.M., F.C., and T.O. analyzed most experimental data; H. Bohnenberger and P.S. performed IHC analyses; T.S. developed and maintained software for single-cell imaging and tracking; J.B., G.A., J.C., A.W., T. Beissbarth, F.S., A.C., N.H., S.G., A.R., L.P., M.R., H. Bönig, C.M.-T., F.K., E.S., A.R.G., H.U., and K.S. contributed and analyzed data, and provided discussion. H.S. provided discussion. F.C., T. Berg, and T.O. wrote the manuscript.

## Supplemental Information

Document S1. Figures S1–S6

Table S1. Proteome and Phosphoproteome of H and H/M Cells, Related to Figures 1 and 2

Table S2. Molecular Characteristics of the AML Patient Cohort, Related to Figures 1, 2, and 5

Document S2. Article plus Supplemental Information
