# Supplementary material for: Hoxa9 and Meis1 Cooperatively Induce Addiction to Syk Signaling by Suppressing miR-146a in Acute Myeloid Leukemia
Source: Cancer Cell. 2017 Apr 10;31(4):549–562.e11. doi: 10.1016/j.ccell.2017.03.001 (PMC5389883; doi:10.1016/j.ccell.2017.03.001)
Supplement: Document S1. Figures S1–S6 [file mmc1.pdf]

Cancer Cell, Volume *31*

Supplemental Information

Hoxa9 and Meis1 Cooperatively Induce Addiction to Syk Signaling by Suppressing miR-146a in Acute Myeloid Leukemia

Sebastian Mohr, Carmen Doebele, Federico Comoglio, Tobias Berg, Julia Beck, Hanibal Bohnenberger, Gabriela Alexe, Jasmin Corso, Philipp Ströbel, Astrid Wachter, Tim Beissbarth, Frank Schnütgen, Anjali Cremer, Nadine Haetscher, Stefanie Göllner, Arefeh Rouhi, Lars Palmqvist, Michael A. Rieger, Timm Schroeder, Halvard Bönig, Carsten Müller-Tidow, Florian Kuchenbauer, Ekkehard Schütz, Anthony R. Green, Henning Urlaub, Kimberly Stegmaier, R. Keith Humphries, Hubert Serve, and Thomas Oellerich

SUPPLEMENTAL DATA

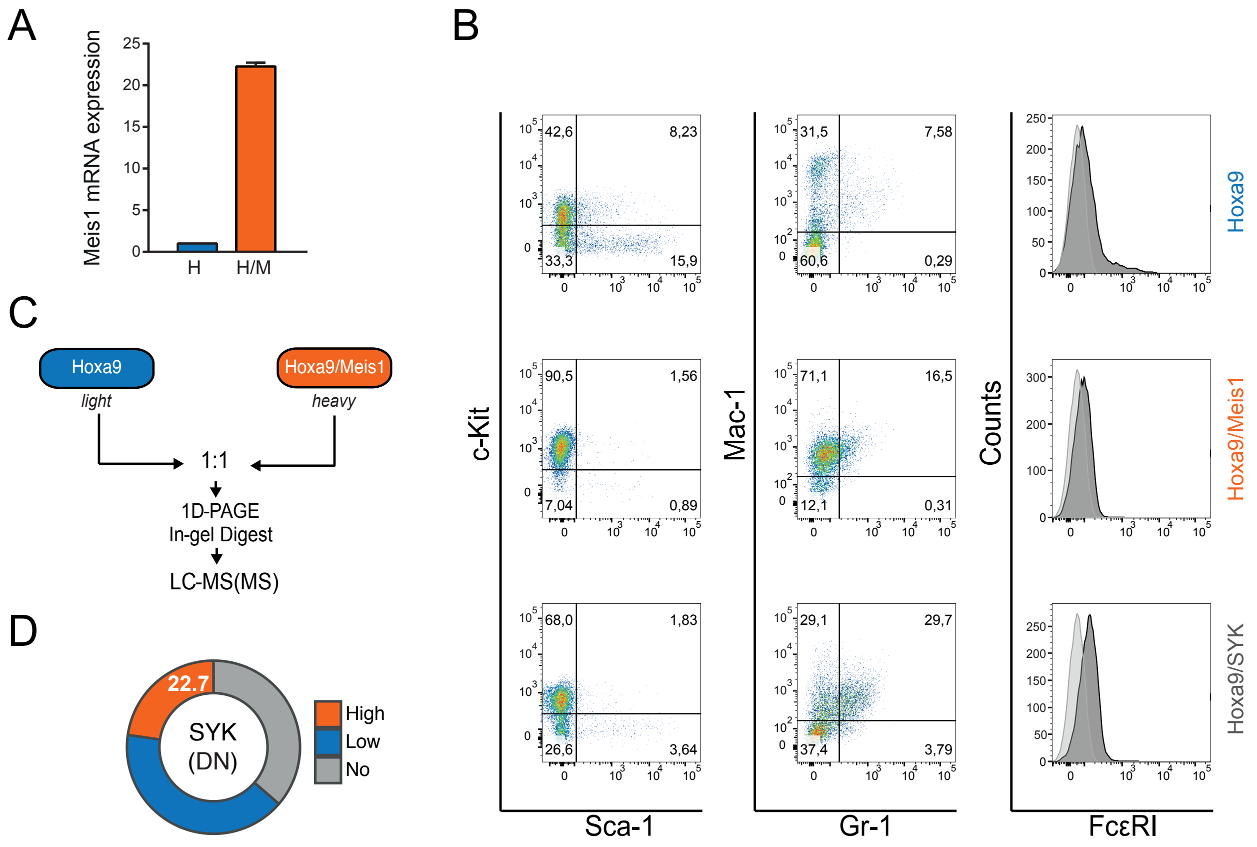

Figure S1. Immunophenotypic and proteomic characterization of H and H/M cells. Related to Figure 1. (A) Relative Meis1 mRNA expression as measured by qPCR, normalized to GAPDH expression (mean±SD, n=3). (B) Immunophenotypic characterization of H, H/M and H/S cells by flow cytometry. (C) Schematic workflow of SILAC labeling of H and H/M cells. H and H/M cells were labeled with light and heavy amino acids, respectively. Lysates were mixed in a 1:1 ratio, proteins were separated by 1D PAGE, digested with trypsin, and analyzed by mass spectrometry (LC-MS/MS). (D) Proportions of SYK expression levels in 66 AML cases with no detectable HOXA9 or MEIS1 overexpression (double negatives, DN) as determined by two independent pathologists using a three-stage staining score.

Table S1. Proteome and phosphoproteome of H and H/M cells. Related to Figures 1 and 2. This table is provided as a separate Excel file.

Table S2. Molecular characteristics of the AML patient cohort. Related to Figures 1, 2 and 5. This table is provided as a separate Excel file.

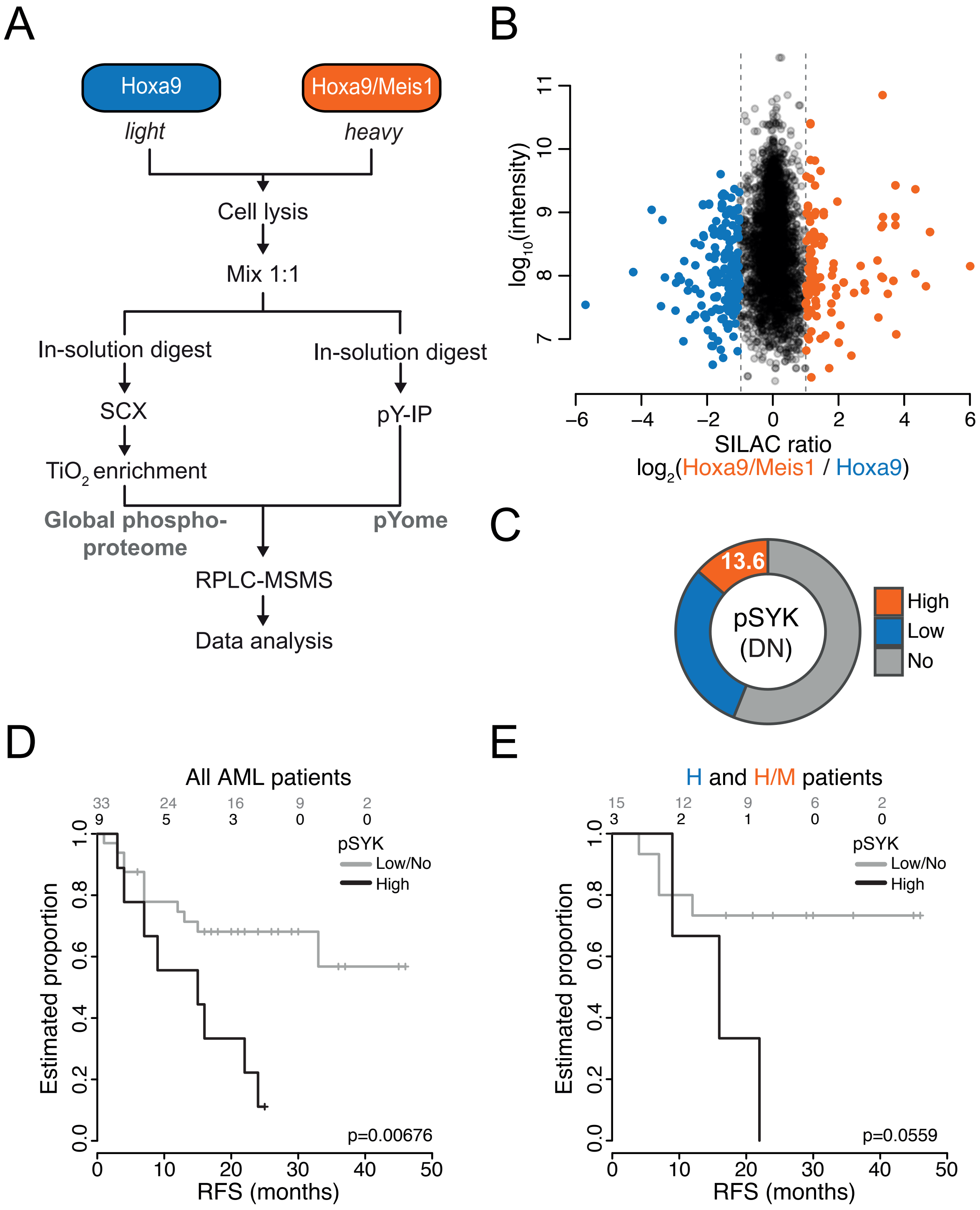

Figure S2. Phosphoproteomic profiling of H and H/M cells, and survival analysis of AML patients. Related to Figure 2. (A) Schematic representation of a SILAC approach for profiling phosphorylation in H and H/M cells. Cells were cultured in SILAC medium as indicated. Lysates were mixed in a 1:1 ratio and proteins were digested with trypsin. Resulting phosphopeptides were enriched by either SCX/TiO_2 (global phosphoproteome analysis; GPome) or phosphotyrosine

immunoprecipitation (pY-IP; pYome analysis), and analyzed by mass spectrometry (LC-MS/MS). (B) Peptide peak intensities versus average normalized SILAC ratios for p-sites identified by a mass-spectrometric GPome analysis in two biological replicates. Blue and orange dots indicate p-sites upregulated in H and H/M cells, respectively. (C) Proportions of pSYK expression levels in 66 AML cases with no detectable HOXA9 or MEIS1 overexpression (double negatives, DN) as determined by two independent pathologists using a three-stage staining score. (D, E) Kaplan-Meier survival analysis for relapse-free survival (RFS) in which all AML patients with complete clinical profiles (D) or H and H/M patients only (E) were grouped by pSYK expression. The number of patients at risk belonging to each category is shown. p value is from a Mantel-Cox test.

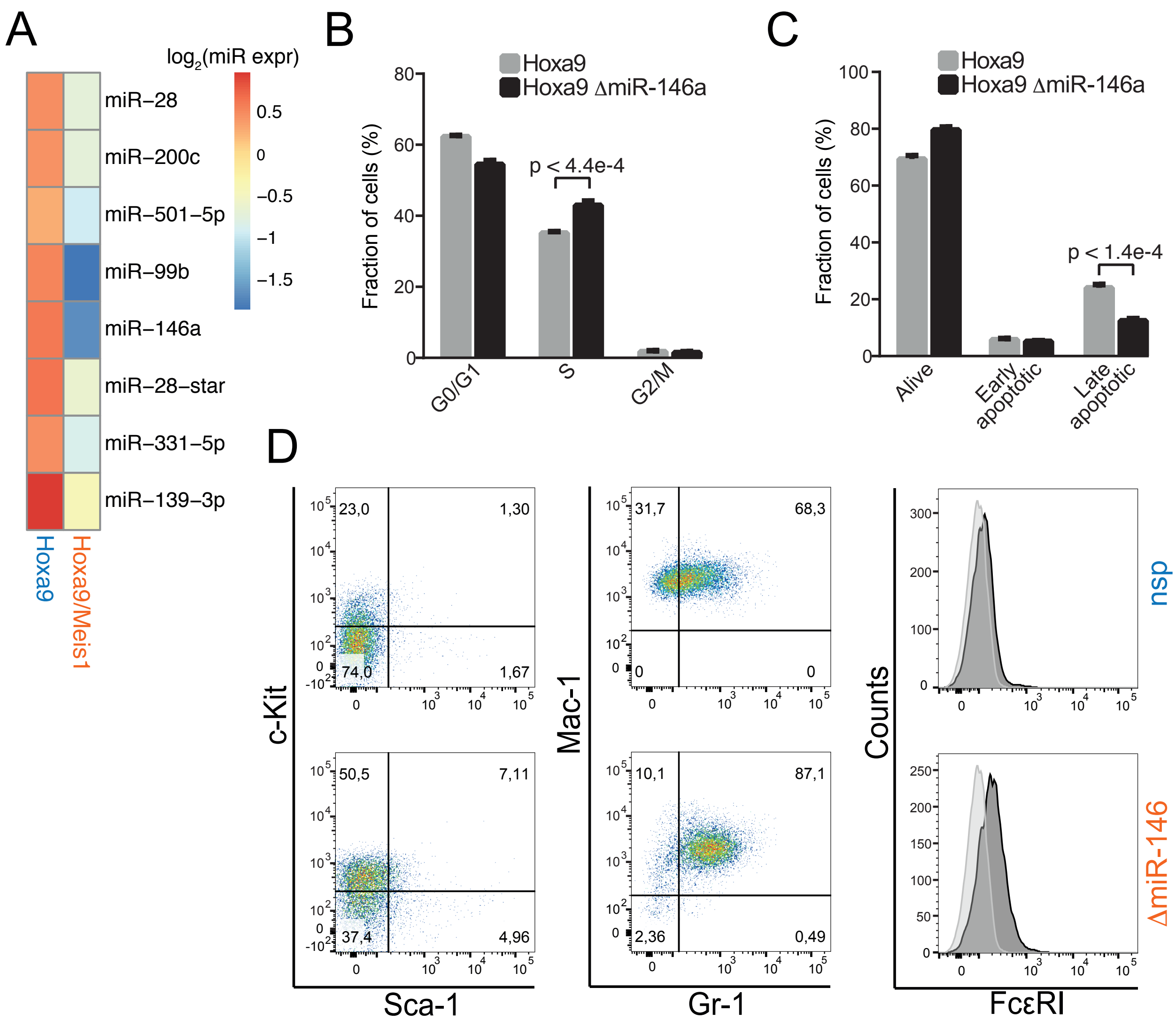

Figure S3. miRNA profiling of H and H/M cells, and characterization of ΔmiR-146 cells. Related to Figure 4. (A) Heatmap of normalized expression values for miRNAs that were significantly downregulated in H/M versus H cells (q<0.01). miRNAs are ranked by decreasing q values, which were derived from a moderated t-test and Benjamini-Hochberg multiple testing correction. (B, C) BrdU incorporation-based cell cycle analysis (B) and AnnexinV/7-AAD-based apoptosis analysis (C) of Hoxa9-transduced BM cells derived from B6.Cg-Mir146$^{tm1.1Bal}$/J knockout mice (ΔmiR-146) (mean±SD, n=3). p values are from a two-sided unpaired t-test. (D) Immunophenotypic characterization of H cells transduced with either a lentiviral non-specific (nsp) control CRISPR or a CRISPR targeting miR-146 (ΔmiR-146).

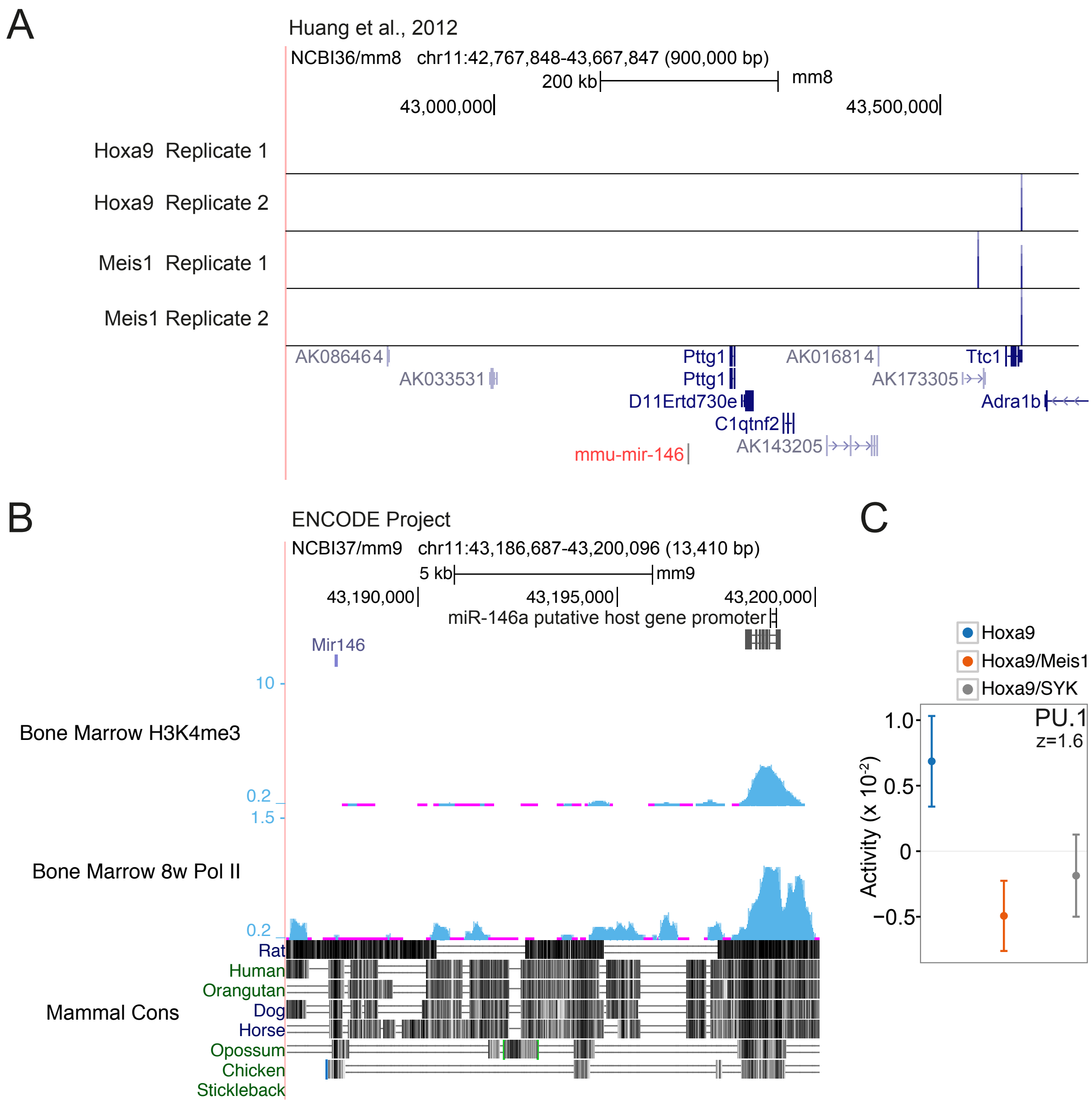

Figure S4. Absence of Meis1 binding in the vicinity of miR-146a. Related to Figure 5. (A) UCSC genome browser shot of a 900 kb region centered on the miR-146a locus. Hoxa9 and Meis1 ChIP-seq profiles were obtained from Huang et al., 2012. (B) UCSC genome browser shot of H3K4me3 and RNA Polymerase II ChIP-seq signals within a 13 kb region centered on the putative miR-146a host gene promoter. The ChIP-seq profiles were generated by Bing Ren's laboratory at the Ludwig Institute for Cancer Research (LICR) and released in ENCODE Release 3 (Aug 2012) (ENCODE Project Consortium, 2012). (C) PU.1 activity profile across H, H/M and H/S cells, inferred by ISMARA using RNA-seq data. The z score of the PU.1 motif is indicated.

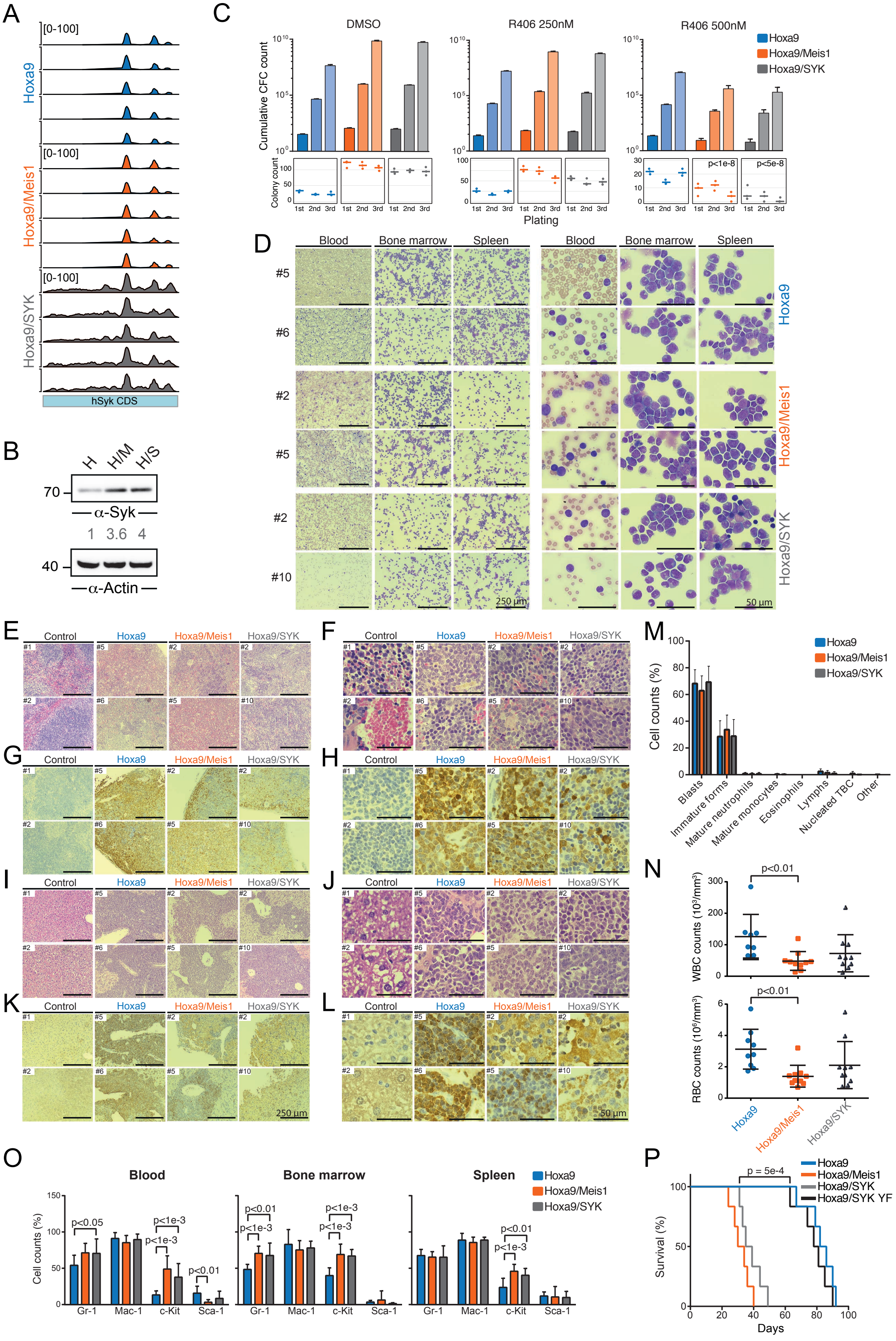

Figure S5. Characterization of H, H/M and H/S leukemias. Related to Figure 6. (A) RNA-seq read coverage throughout the human SYK coding sequence. (B) Syk protein expression in H, H/M and H/S cells by immunoblotting. Actin was used as loading control for relative protein quantification. (C) (top) Cumulative colony-forming cell (CFC) count for an initial plating of 100 cells transformed by Hoxa9 alone or in combination with Meis1 or SYK and treated with either DMSO or R406, for three sequential rounds of plating. (bottom) Corresponding colony count (mean$\pm$SD, n=3). p values are from a repeated measures ANOVA. (D) Morphology of developing leukemias. Blood smears and cytospins of bone marrow and spleen were stained by Pappenheim's staining and are shown for comparison from two individual samples for Hoxa9, Hoxa9/Meis1 and Hoxa9/SYK (left, scale bar: 250 μm; right, scale bar: 50 μm). (E, F) Haematoxylin and eosin staining of spleens from two individual samples for control, H, H/M and H/S cells (E, scale bar: 250 μm; F, scale bar: 50 μm). (G, H) Immunohistochemistry staining for YFP of spleens from two individual samples for control, H, H/M and H/S cells (G, scale bar: 250 μm; H, scale bar: 50 μm). (I, J) Haematoxylin and eosin staining of liver from two individual samples for H, H/M and H/S cells. (I, scale bar: 250 μm; J, scale bar: 50 μm) (K, L) Immunohistochemistry staining for YFP of liver from two individual samples for control, H, H/M and H/S cells (K, scale bar: 250 μm; L, scale bar: 50 μm). (M) Cell counts from cytospins of bone marrow stained by Pappenheim's staining from H (n=9), H/M (n=10) and H/S (n=10) mice. Values are mean$\pm$SD. (N) White blood count (top) and red blood count (bottom) from mice with H (n=8), H/M (n=10) and H/S (n=10) induced leukemias. Values are mean$\pm$SD. p values are from a two-sided unpaired t-test. (O) Surface marker expression of H (n=9), H/M (n=10) and H/S (n=10) leukemia cells from transplanted mice as measured by flow cytometry. Values are mean$\pm$SD. p values are from a two-sided unpaired t-test. (P) Kaplan-Meier survival curves of mice transplanted with H, H/M or H/S cells, or with H cells expressing a hSYK Y348F/Y352F double mutant (n=6). p value is from a Mantel-Cox test.

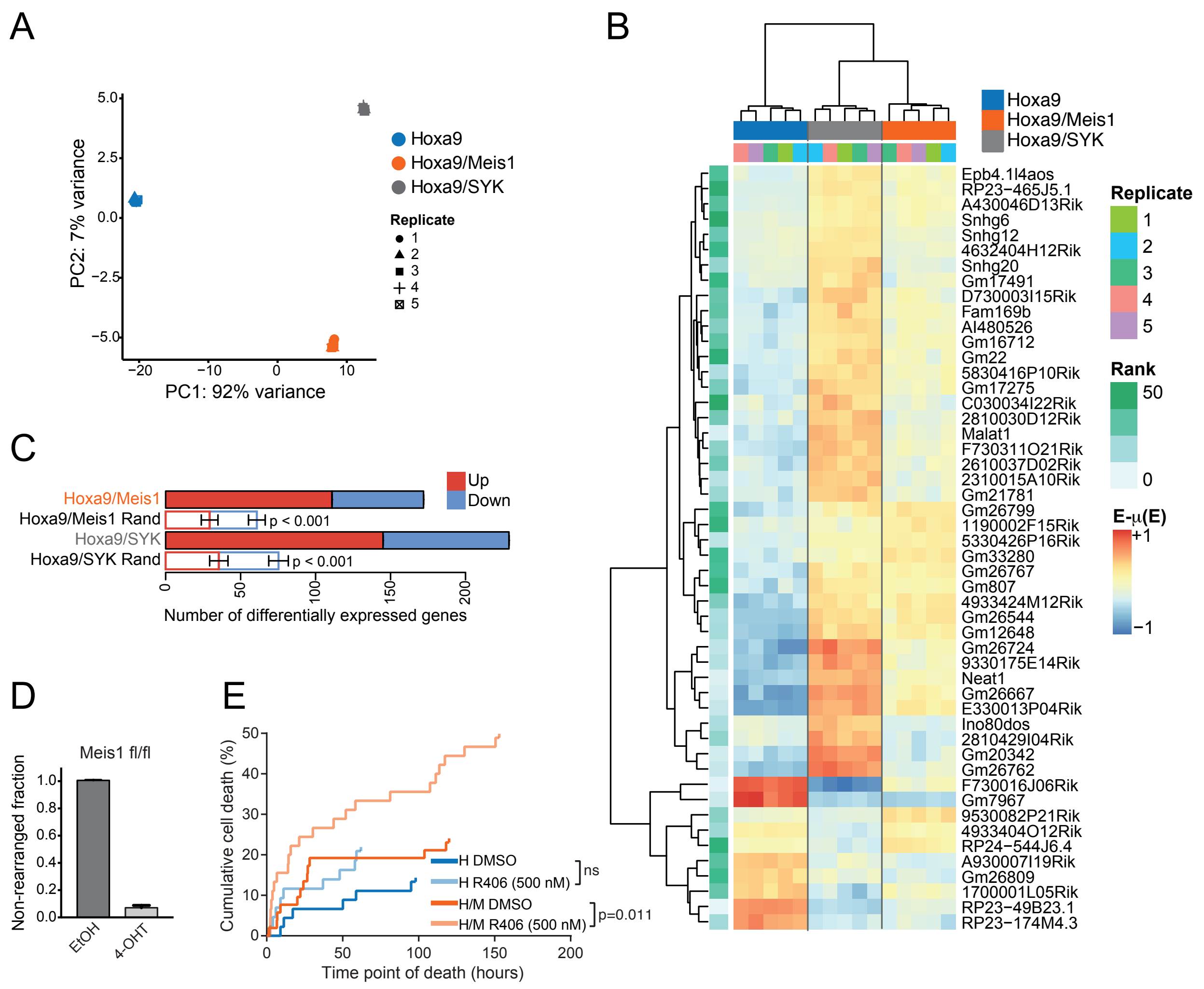

Figure S6. Transcriptional profiling of H, H/M and H/S cells, and effect of Syk inhibition. Related to Figure 7. (A) Principal component analysis of H, H/M and H/S gene expression. Genes with zero counts across all samples were excluded from the analysis. (B) Hierarchical clustering of the top 50 differentially expressed lincRNAs (adjusted p-value $\leq 10^{-5}$, likelihood ratio test). (C) Number of differentially expressed genes bound by Meis1 within 5kb from their transcription start sites. Empirical expectations were computed by Meis1 peak shuffling (1000 simulations) and empirical p values are indicated. (D) Fraction of non-rearranged H/S cells derived from inducible Meis1 knockout mice. Cells were treated with either ethanol (EtOH, control) or 4-hydroxytamoxifen (4-OHT). Values are mean±SD (n=3). (E) Cumulative cell death and time point of death upon Syk inhibitor treatment assessed by videomicroscopy-based cell tracking at single cell resolution. For each curve, the last indicated time point corresponds to the last cell death event. p values are from a two-sided Mann-Whitney U test; ns, not significant.
